# Proteins in human body fluids contain in vivo antigen analog of the melibiose-derived glycation product: MAGE

**DOI:** 10.1038/s41598-022-11638-2

**Published:** 2022-05-07

**Authors:** Kinga Gostomska-Pampuch, Andrzej Gamian, Karol Rawicz-Pruszyński, Katarzyna Gęca, Joanna Tkaczuk-Włach, Ilona Jonik, Kinga Ożga, Magdalena Staniszewska

**Affiliations:** 1 Department of Biochemistry and Immunochemistry, Wroclaw Medical University, Chalubinskiego 10, 50-368 Wrocław, Poland; 2 Laboratory of Medical Microbiology, Ludwik Hirszfeld Institute of Immunology and Experimental Therapy, Polish Academy of Sciences, Weigla 12, 53-114 Wrocław, Poland; 3 Department of Surgical Oncology, Medical University of Lublin, Radziwillowska 13, 20-080 Lublin, Poland; 4 Diagnostic Techniques Unit, Collegium Maximum, Medical University of Lublin, Staszica 4/6, 20-081 Lublin, Poland; 5 Faculty of Science and Health, The John Paul II Catholic University of Lublin, Konstantynów 1J, 20-708 Lublin, Poland

**Keywords:** Blood proteins, Chemical modification, Gastric cancer, Biomarkers

## Abstract

Melibiose-derived AGE (MAGE) is an advanced glycation end-product formed in vitro in anhydrous conditions on proteins and protein-free amino acids during glycation with melibiose. Our previous studies revealed the presence of MAGE antigen in the human body and tissues of several other species, including muscles, fat, extracellular matrix, and blood. MAGE is also antigenic and induces generation of anti-MAGE antibody. The aim of this paper was to identify the proteins modified by MAGE present in human body fluids, such as serum, plasma, and peritoneal fluids. The protein-bound MAGE formed in vivo has been isolated from human blood using affinity chromatography on the resin with an immobilized anti-MAGE monoclonal antibody. Using mass spectrometry and immunochemistry it has been established that MAGE epitope is present on several human blood proteins including serum albumin, IgG, and IgA. In serum of diabetic patients, mainly the albumin and IgG were modified by MAGE, while in healthy subjects IgG and IgA carried this modification, suggesting the novel AGE can impact protein structure, contribute to auto-immunogenicity, and affect function of immunoglobulins. Some proteins in peritoneal fluid from cancer patients modified with MAGE were also observed and it indicates a potential role of MAGE in cancer.

## Introduction

Advanced glycation end-products (AGEs) form during glycation and accumulate in a body disturbing protein and tissue homeostasis^1^. AGEs constitute a group of antigens that can induce generation of the autoantibodies^2,3^. Recently, our group has revealed presence of the novel unconventional AGE antigen accumulating in different tissues of various species. This is a structural analog of the product called MAGE that forms from melibiose (mel) during an in vitro protein glycation in anhydrous conditions. The structure of a low-molecular MAGE product (protein-free) determined by NMR analysis showed a mixture of isomers with an open chain and a cyclic form of the fructosamine moiety. Using the model MAGE we have also shown that the serum from diabetic patients contains the autoantibody specifically binding MAGE^4^. Immunohistochemical analyzes with the in-house generated anti-MAGE antibody have shown that the novel AGE structural analog accumulates in a variety of human and animal tissues. An intense staining was observed on slides with sections from skeletal muscle of human, horse, pig, frog, and fish, in cardiomyocytes of pig, chicken and rat, and in smooth muscle of pig and rat. In addition, MAGE has been detected in the extracellular matrix of various animal tissues^4^, including various cancer tumors (manuscript in preparation). MAGE has also been associated with diabetic complications and atherosclerosis^5,6^. Interestingly, in other studies MAGE showed genotoxicity on human peripheral blood lymphocytes, melanoma, lung cancer, and colorectal cancer cells in vitro, while protein-free adducts prevented cells from this effect^7^. This observation indicates a potential role of MAGE in vivo, however the origin, detailed structure, and specific effect in different tissues remain to be elucidated. It should be noted that melibiose is produced during food fermentation by bacteria of the genus *Bifidobacterium*^8,9^, *Lactobacillus*, *Lactococcus, Leuconostoc*^10^, and yeast^11^. It is also found in cocoa beans, honey, and processed soybeans^12,13^. Supplied with food, melibiose is absorbed in the small intestine through para-cell junctions existing between adjacent enterocytes^14^. This suggests an exogenous source of mel as a substrate for further MAGE production. However, there are currently no data available on possible MAGE formation in the gut and subsequent absorption into the bloodstream.

Glycation occurs on various molecules in the body, including proteins in the circulatory system and solid tissues on the extra- and intracellular proteins. The formation of AGE adducts is favored on the long-lived proteins. This group of proteins includes lens crystallins^15^, collagen, blood albumin^16^, insulin, immunoglobulins^17^, and low-density lipoproteins (LDL)^18–21^. Glycation of insulin in pancreatic cells or blood makes it difficult to maintain homeostatic glucose levels and stimulate lipogenesis^22^. Glycated albumin and methylglyoxal (an intermediate glycation metabolite) can enhance insulin resistance, e.g. by blocking the PI3K/Akt signaling pathway and inducing oxidative stress, which leads to a strong inhibition of metabolic processes regulated by this hormone^23,24^. These factors contribute to the chronic hyperglycemia observed in type 2 diabetes, which in turn enhances protein glycation^19^. In addition, a high content of glycated albumin in the blood may disturb immune balance and induce oxidative stress, causing tissue inflammation^25^. This process can also have a detrimental effect on the immune system as glycation of immunoglobulins impacts protein function^19^. Glycated LDL proteins interact with scavenger receptors on the surface of macrophages causing differentiation into foam cells and atherosclerotic plaques^26^. Moreover, glycation provides proteins with immunogenic properties and causes their accumulation in plasma in form of immune complexes^19,27^. In the extracellular matrix and connective tissue, collagen, laminin, vitronectin, and elastin are targets for glycation. Such modifications result in increased stiffening of the walls of blood vessels or heart muscle, cause cardiovascular complications (especially in diabetic patients), and lead to the development of angiopathy, retinopathy, nephropathy, neuropathy, and cardiomyopathy^1,28–31^.

AGEs can also interact with some specific receptors and excerpt cellular effects. The best known receptor—RAGE has been identified on the surface of various types of cells and tissues, e.g. on macrophages, phagocytes, neutrophils, hepatocytes, endothelial smooth muscle, nervous system, and mesangial cells^1,32^. Under physiological conditions, RAGE is expressed at a low level, but elevated concentration of ligands (including AGEs) causes receptor overexpression in pathological or chronic inflammation associated with diabetes, Alzheimer's and cardiovascular disease or cancer^33^. RAGE interaction with the ligands initiates signaling pathways through the mitogen-activated protein kinases (MAPKs), such as p44/42 (ERK1/2), p38, and JNK^34^. In effect activation of NF-κB leads to upregulation of Jak/STAT and p21^ras^ pathways, AKT kinases, GSK-3β, and rac-1 molecules^35^ responsible for several effects, i.e. inflammation, cell proliferation, angiogenesis, fibrosis, thrombogenesis, and apoptosis^32^. This creates pro-inflammatory conditions with release of cytokines Il-1 (interleukin-1), Il-6, and TNF-α (tumor necrosis factor)^36^. Moreover, the AGEs—RAGE interaction can induce NADPH oxidase and oxidative stress through reactive oxygen species (ROS), which also affect induction of the NFκB^37,38^. The increased production of ROS and the dysregulated inflammatory process largely contribute to cancer formation^38^. AGEs were shown to play a role in proliferation, migration, invasion, and/or increased angiogenic potential of cancer cells^39^. The tumor-promoting effects of AGEs have been revealed in studies on several cancer, like colon^40^, prostate^41^, lung^42^, liver^43,44^, breast^45^, pancreatic^46,47^, and melanoma^48^. It has been reported that AGEs derived from glucose are present in cancer tissues and blood of gastric cancer (GC) patients. Interestingly, RAGE expression is increased in in invasive cancer compared to the non-invasive tumor and it is associated with disease progression. Therefore, in invasive cancer the accumulation of glucose-derived AGEs and RAGE expression is associated with the potential risk for disease progression^49^. However, the effect of MAGE on cancer microenvironment and its role in RAGE-mediated cellular response remain to be discovered.

To date, only few AGEs have been characterized and confirmed in vivo, with many other perhaps crucial for a specific pathology, are yet to be described. While the structure and basic biological properties of the in vitro obtained MAGE has been elucidated^4,7^, there is need for determination of the natural in vivo counterpart. The goal of the presented paper was to identify the proteins modified by MAGE present in human body fluids, especially in blood and peritoneal fluid from healthy, diabetic and cancer individuals. We present the isolation of natural MAGE antigen, identification of the type of carrier protein, and characterization by means of spectrometric and immunochemical methods. We also indicate the presence of the MAGE antigen on proteins derived from blood and peritoneum in gastric cancer (GC) patients. Our data initiate an interesting perspective on MAGE in cancer microenvironment.

## Results

### Identification of blood proteins reacting with anti-MAGE antibody

**Figure 1:**
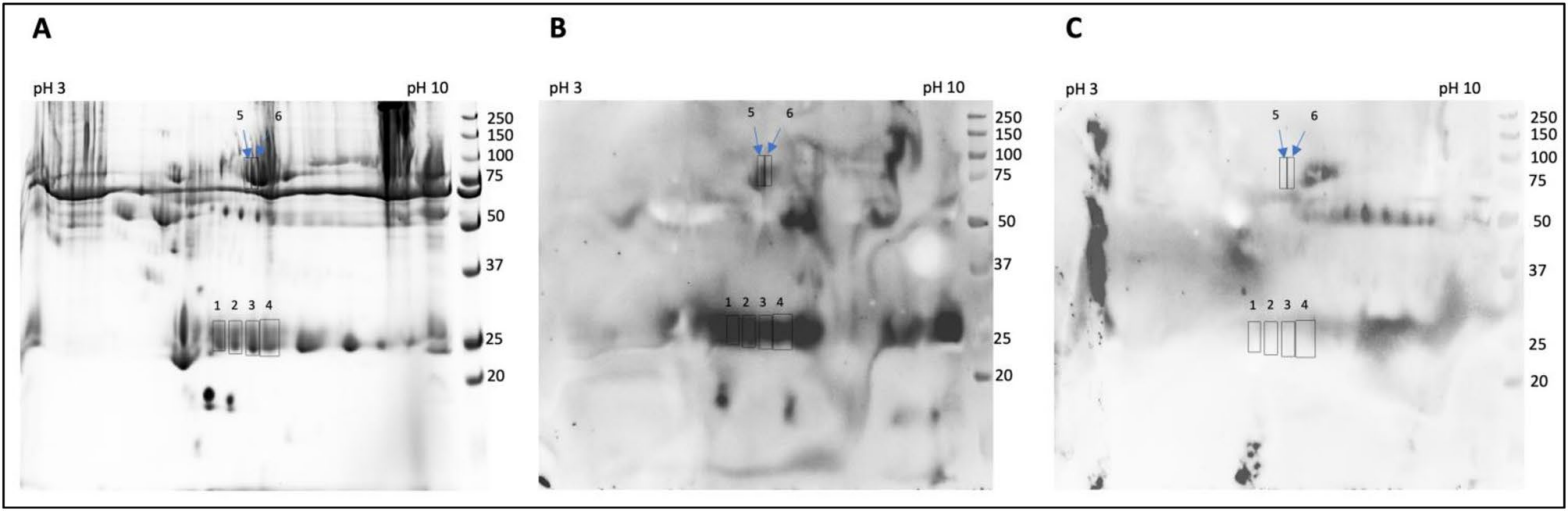
Identification of proteins glycated with MAGE. Protein from human serum were subjected to isoelectric focusing between pH 3–7, separated on 8% SDS-PAGE gel and stained with Coomassie Safe (**A**). Additional gels were subjected to WB analysis with anti-MAGE mAb followed by secondary Ab anti-mouse IgE-HRP (**B**) or by incubation with the secondary Ab only (**C**). The indicated spots 1–6 were excised from the gel. Molecular mass markers on the right side of each panel indicate mass in kDa. The original gel and blots are presented in Supplementary Fig. S4.

The proteins present in serum sample obtained from a healthy volunteer were at first separated by 2D electrophoresis (Fig. 1A) and subjected to a Western Blotting (WB) analysis to identify MAGE modifications. Staining with the specific anti-MAGE monoclonal antibody (mAb) revealed several proteins caring the MAGE antigen (Fig. 1B). Some of the spots, i.e. proteins with ~ 25 kDa (spot 1–4) and ~ 75 kDa (5–6) were selected for protein identification using mass spectrometry analysis.

Several proteins have been identified in each of the excised samples (spot 1–6), although the employed criteria were very stringent, included hits with score > 1000 and high sequence coverage (Table 1). The spots 1–4 that in 2D gel migrated as the proteins around 25 kDa, revealed presence of the light chain of immunoglobulin kappa and lambda, while the spot 5 and 6 migrating as proteins around 75 kDa showed several proteins including serum albumin, heavy chain of immunoglobulin mu and gamma. These results suggest that the common and highly abundant blood proteins can be modified with MAGE. Presence of other proteins, especially with unrelated molecular mass (spot 1–3—albumin, spot 3—serotransferrin, spot 5—complement C3) needs further explanation and verification.

**Table 1:** The list of proteins in the individual gel spots identified by mass spectrometry.

Spot	No. in sprot database	Protein (score > 1000)	Score	Mass (Da)	Number of matched peptides	Sequence coverage (%)
1	P0DOX7	Immunoglobulin kappa light chain	9428	23,650	223	58
	P01834	Immunoglobulin kappa constant	9287	11,929	236	84
	P0DOY2	Immunoglobulin lambda constant 2	4307	11,458	156	74
	P0DOX8	Immunoglobulin lambda-1 light chain	3118	11,430	105	38
	P02768	Albumin	2450	71,317	68	49
	A0M8Q6	Immunoglobulin lambda constant 7	1874	11,418	52	41
	P02647	Apolipoprotein A	1288	30,759	32	62
2	P01834	Immunoglobulin kappa constant	10,177	11,929	261	84
	P0DOX7	Immunoglobulin kappa light chain	10,078	23,650	232	58
	P0DOY2	Immunoglobulin lambda constant 2	3423	11,458	139	93
	P0DOX8	Immunoglobulin lambda-1 light chain	2190	23,101	139	51
	P02768	Albumin	2110	71,317	68	50
	P02647	Apolipoprotein A	2026	30,759	53	70
	P01876	Immunoglobulin heavy constant alpha 1	1090	38,486	25	36
3	P0DOX7	Immunoglobulin kappa light chain	14,063	23,650	310	57
	P01834	Immunoglobulin kappa constant	13,761	11,929	319	82
	P0DOY3	Immunoglobulin lambda constant 3	3668	11,430	145	84
	P0DOX8	Immunoglobulin lambda-1 light chain	2471	23,101	84	47
	P02768	Albumin	2457	71,317	68	62
	P02647 ara>	Apolipoprotein A	1872	30,759	45	68
	P01619	Immunoglobulin kappa variable 3–20	1627	12,663	24	27
	A0A0C4DH25	Immunoglobulin kappa variable 3D-20	1520	12,621	22	27
	A0M8Q6	Immunoglobulin lambda constant 7	1370	11,418	41	41
	P02787	Serotransferrin	1013	79,294	35	28
4	P01834	Immunoglobulin kappa constant	15,616	11,929	340	82
	P0DOX7	Immunoglobulin kappa light chain	15,451	23,650	318	57
	P0DOY2	Immunoglobulin lambda constant 2	4297	11,458	165	93
	P02768	Albumin	4132	71,317	107	59
	P02647	Apolipoprotein A	2920	30,759	62	67
	P0DOX8	Immunoglobulin lambda-1 light chain	2568	23,101	95	47
	P01619	Immunoglobulin kappa variable 3–20	2517	12,663	37	51
	A0A0C4DH25	Immunoglobulin kappa variable 3D-20	2322	12,621	31	27
	P02787	Serotransferrin	1693	79,294	46	33
	P0DOX5	Immunoglobulin gamma-1 heavy chain	1336	49,925	29	33
	P01859	Immunoglobulin heavy constant gamma 2	1065	36,505	25	29
5	P02787	Serotransferrin	18,750	79,294	479	61
	P02768	Albumin	10,758	71,317	302	72
	P01871	Immunoglobulin heavy constant mu	1901	50,093	51	37
	P00734	Prothrombin	1887	71,475	36	26
	P01008	Antithrombin-III	1717	53,025	41	42
	P01024	Complement C3	1311	188,569	32	12
	P02790	Hemopexin	1198	52,385	33	35
	P0DOX5	Immunoglobulin gamma-1 heavy chain	1083	49,925	25	28
	P01876	Immunoglobulin heavy constant alpha 1	1046	36,596	24	36
	P01834	Immunoglobulin kappa constant	1025	11,929	18	79
6	P02787	Serotransferrin	22,934	79,294	628	74
	P02768	Albumin	12,611	71,317	351	76
	P01871	Immunoglobulin heavy constant mu	1089	50,093	31	30
	P02790	Hemopexin	1070	52,385	28	36
	P0DOX5	Immunoglobulin gamma-1 heavy chain	914	49,925	22	31

*Keratin was identified in samples but not considered here.

### Isolation of MAGE analog from human blood

**Figure 2:**
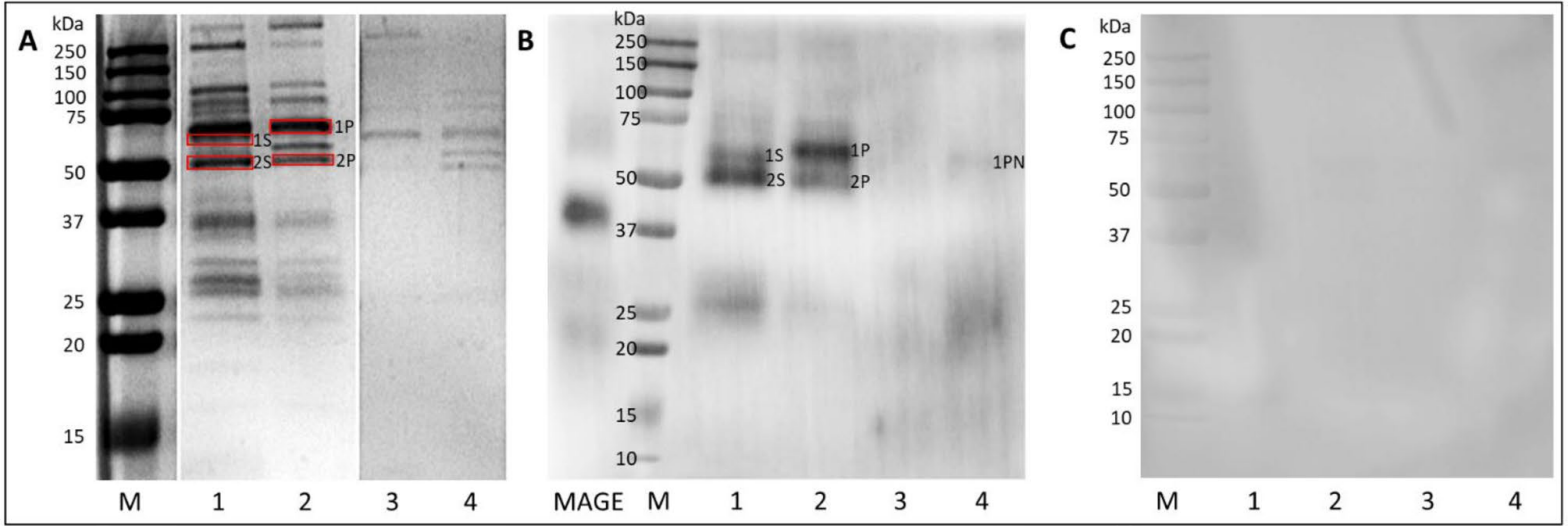
Extraction of blood proteins glycated with MAGE. Proteins from serum of the diabetic patient and plasma of healthy donor were immunoprecipitated with the Sepharose-anti-MAGE resin and the same volume of each sample was analyzed by SDS-PAGE (**A**). The separated in the gel proteins that bound to the Sepharose-anti-MAGE antibody resin (lane 1, 2) and the control resin (lane 3, 4) were stained with Coomassie Brilliant Blue or were subjected to WB with the anti-MAGE antibody (**B**). As the negative control, the additional membrane was probed with the secondary anti-mouse IgE-HRP antibody (**C**). The proteins indicated in panel A by red boxes corresponding to bands 1S, 2S (diabetic serum) and 1P, 2P (plasma from healthy donor) showed specific binding with the anti-MAGE antibody and were excised from gel for mass spectrometry analysis. M—molecular mass standard; MAGE (MB-mel). The original gel and blots are presented in Supplementary Fig. S5.

After an initial 2D electrophoresis tracking of the blood proteins modified by MAGE the antigen has been extracted by employing an immunoprecipitation. The purified anti-MAGE monoclonal antibody (Supplementary Figs. S1, S2) and Pierce Direct IP Kit (Thermo Scientific, Waltham, MA, USA) were used in accordance with the manufacturer's instructions. Serum samples from the diabetic patients was applied to columns with the immobilized anti-MAGE antibody. The same experiment was performed with a human blood plasma from the healthy donors. The material bound to the Sepharose-anti-MAGE resin was analyzed by SDS-PAGE and showed number of proteins (Fig. 2A, lane 1 and 2). Some of the proteins were also eluted from the control resin without anti-MAGE antibody (Fig. 2A, lane 3 and 4). However, in this case only few proteins showed WB staining with anti-MAGE antibody, i.e. 59.4 kDa (1S) and 50.0 kDa (2S) (Fig. 2B; lane 1) in a blood of the diabetic patients, and proteins of 61.7 kDa (1P) and 49.3 kDa (2P) (Fig. 2B; lane 2) in a blood of the healthy donor. Some weak reaction was evident in the sample eluted from the control resin (1PN, Fig. 2B; lane 4), however the mass of this protein (56.7 kDa) was different than the protein eluted from Sepharose-anti-MAGE resin (Fig. 2B, lane 1, 2), suggesting the non-specific binding of this blood protein to the resin not present in sample 2. The non-specific secondary antibody reaction in WB was excluded by staining of the membrane with the secondary antibody only (Fig. 2C). The gel bands (marked in red in Fig. 2A) corresponding to the proteins labeled with the anti-MAGE antibody (Fig. 2B, lane 1, 2) were excised and subjected to the mass spectrometry protein identification.

Mass spectrometry analysis of the excised protein bands revealed several proteins in the analyzed samples (Table 2). In the sample from diabetic patients (1S), the strongest signal (score) and sequence coverage of 38.8% was shown for albumin, α-1-antitrypsin, and heavy chain of immunoglobulin A (IgA) and G (IgG). In the 2S sample, IgG1 heavy chain (30% sequence coverage) and the constant regions of the IgG3 and IgG2 heavy chains were identified. The sample 1P from the healthy donor plasma contained fibrinogen α-chain as the protein with the strongest signal, although sequence coverage was slightly greater for the heavy chain of IgA (20.4%). In the 2P sample, the strongest signal was obtained for the heavy chain of IgG1 (28.7% sequence coverage). In this sample the presence of IgG2 heavy chain as well as β and γ fibrinogen was also found.

**Table 2:** The list of proteins glycated by MAGE extracted from human blood and identified by mass spectrometry.

Band	No. in sprot database	Protein (score > 350)	Score	Mass (Da)	Number of matched peptides	Sequence coverage (%)
1S	P02768	**Albumin**	2900	71,317	64	38.8
	P01009	**Alpha-1-antitrypsin**	1510	46,878	31	41
	P01876	**Immunoglobulin heavy constant alpha 1**	1495	38,486	29	24
	P01860	**Immunoglobulin heavy constant gamma 3**	1453	42,287	35	21
	P0DOX5	**Immunoglobulin gamma-1 heavy chain**	1406	49,925	33	16
	P01877	**Immunoglobulin heavy constant alpha 2**	1077	37,366	22	23
	P0DOX2	Immunoglobulin alpha-2 heavy chain	956	49,816	21	19
	P01859	Immunoglobulin heavy constant gamma 2	891	36,505	26	19
	P04004	Vitronectin	758	55,069	15	19
	P08670	Vimentin	620	53,676	5	4
	P01008	Antithrombin-III	619	53,025	13	23
	P02765	Alpha-2-HS-glycoprotein	539	40,114	9	11
	P01861	Immunoglobulin heavy constant gamma 4	492	36,431	9	13
2S	P0DOX5	**Immunoglobulin gamma-1 heavy chain**	1961	49,925	46	30
	P01857	**Immunoglobulin heavy constant gamma 1**	1961	36,596	46	41
	P01860	**Immunoglobulin heavy constant gamma 3**	1671	42,287	42	18
	P01859	**Immunoglobulin heavy constant gamma 2**	1396	36,505	37	19
	P08670	Vimentin	900	53,676	7	4
	P01861	Immunoglobulin heavy constant gamma 4	523	36,431	11	20 ara>
	P08238	Heat shock protein HSP 90-beta	357	83,554	4	5
1P	P02671	**Fibrinogen alpha chain**	992	95,656	26	19.9
	P08670	Vimentin	752	53,676	7	4.7
	P08238	Heat shock protein HSP 90-beta	431	83,554	7	8.6
	P02768	Albumin	408	71,317	12	14.9
	P01876	**Immunoglobulin heavy constant alpha 1**	352	38,486	9	20.4
2P	P0DOX5	**Immunoglobulin gamma-1 heavy chain**	2510	49,925	52	28.7
	P01859	**Immunoglobulin heavy constant gamma 2**	2297	36,505	55	34
	P02675	**Fibrinogen beta chain**	2127	56,577	52	46.6
	P02679	**Fibrinogen gamma chain**	2112	52,106	37	40.8
	P08670	Vimentin	1025	53,676	9	4.7
	P01861	Immunoglobulin heavy constant gamma 4	790	36,431	14	25.7
	P02671	Fibrinogen alpha chain	637	95,656	19	19.2
	P01871	Immunoglobulin heavy constant mu	398	50,093	5	9.5
	P06733	Alpha-enolase	360	47,481	3	4.6

*S* serum from diabetic patients, *P* plasma from a healthy donor. The identified proteins with the strongest signal and sequence coverage are in bold; Keratin was present but not considered here.

**Figure 3:**
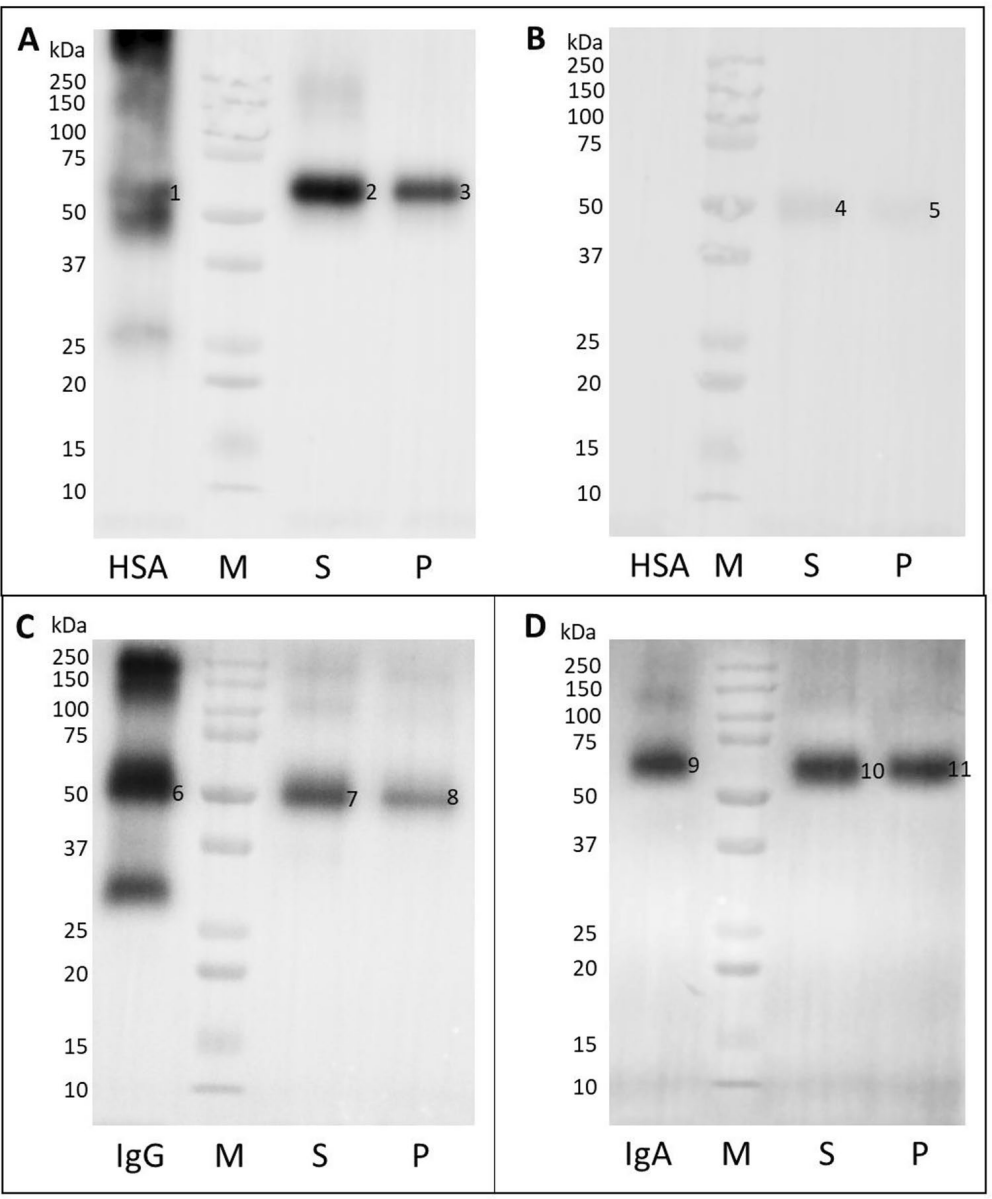
Verification of proteins extracted from human blood by WB analysis. The proteins extracted from diabetic patient serum (S), plasma of healthy donor (P) were transferred on the PVDF membrane along with protein marker (M), HSA, IgG, IgA (2 µg/well) and were probed with anti-HSA (**A**)**,** anti-mouse IgG-HRP (**B**)**,** anti-human IgG-HRP (**C**), and anti-human IgA-HRP (**D**) antibodies. The original blots are presented in Supplementary Fig. S6.

Based on the identification score, sequence coverage (Table 2), and theoretical protein mass the most common proteins in human blood, i.e. albumin (HSA), IgG, and IgA were selected for further consideration. To verify the mass spectrometry results we performed a series of additional WB experiments with specific anti-HSA, anti-human IgG, and anti-human IgA antibodies (Fig. 3). The molecular mass of the identified protein bands was determined using the LabImage software (Kapelan Bio-Imaging, Leipzig, Germany) and were summarized in Table 3.

**Table 3:**
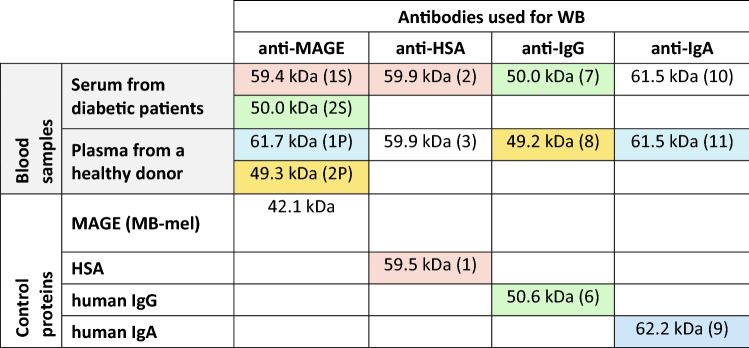
The summary of results obtained by WB of human blood samples or standard proteins (MAGE, HSA, IgG, IgA) and estimation of molecular mass [kDa] of the bands (indicated in brackets) from Figs. 2 and 3.

Colors indicate the corresponding protein bands detected with different antibodies anti-MAGE, anti-HSA, anti-IgG or anti-IgA.

The samples obtained from serum and plasma were probed by WB with the anti-HSA antibody showed the presence of proteins with a mass of ~ 59 kDa (Fig. 3A; S-band 2, P-band 3), that migrated like HSA protein loaded as a control (Fig. 3A; HAS-band 1). On the membrane probed with the anti-mouse IgG-HRP antibody (negative control), the weak band of about 49 kDa was observed (Fig. 3B; bands 4, 5), that presents a non-specific cross-reactivity of the secondary antibody to human IgG present in the samples and proves the HSA identity in the studied samples.

To verify presence of human IgG in the extracted from human blood MAGE protein, the membrane was incubated with the anti-human IgG-HRP antibodies. It showed the proteins with molecular mass of about 49–50 kDa (Fig. 3C; S-band 7, P-band 8) that migrated like IgG loaded as a control (Fig. 3C; IgG-band 6).

Similarly, the reactivity of the extracted proteins with anti-human IgA-HRP antibody showed the protein band of about 61 kDa (Fig. 3D; S-band 10, P-band 11) that migrated similarly to IgA loaded as a control (Fig. 3D; IgA-band 9). The results suggest that the in vivo MAGE antigen extracted from human blood is present on several proteins including HSA (59 kDa), IgG (50 kDa), and IgA (61 kDa).

The results of WB analysis and the molecular mass estimation, based on comparison with the marker proteins, have been summarized in Table 3. Protein band 1S with mass of 59.4 kDa labeled with anti-MAGE antibody (Fig. 2B, lane 1) corresponds to the mass of band 2 in Fig. 3A, lane S, suggesting it is HSA. This has also been confirmed by mass spectrometry analysis of the sample 1S that we identified to be a human albumin protein (Table 2). Bands 2S and 2P (Fig. 3B) with mass of 50.0 and 49.3 kDa, respectively, correspond to bands 7 and 8 (Fig. 3C) detected by the WB with anti-IgG antibodies. These results have been also confirmed by mass spectrometry that identified human IgG in samples 2S and 2P (Table 2). Band 1P (Fig. 2B, lane 2) of 61.7 kDa labeled in WB with anti-MAGE antibody corresponds to band 11 (61.5 kDa) in Fig. 3D and confirms it is IgA. Mass spectrometry analysis also indicated the presence of IgA in sample 1P (Table 2).

Our results suggest the in vivo MAGE antigen extracted from human blood is present on several proteins including HSA (59 kDa), IgG (50 kDa), and IgA (61 kDa). Interestingly, in these preliminary experiments we noticed different proteins modified by MAGE in the tested samples from the diabetic patients comparing to the healthy people. Serum albumin and IgG were the major proteins found to contain MAGE antigen in diabetic serum in contrast to healthy people that plasma showed mostly immunoglobulins G and A to be modified. In addition, the sample after immunoprecipitation with the Sepharose-anti-MAGE resin contained other proteins identified by mass spectrometry (α-1-antitrypsin, vitronectin, vimentin, antithrombin or fibrinogen; Table 2), however they need further verification.

### Analysis of body fluid samples for presence of MAGE-modified proteins

**Figure 4:**
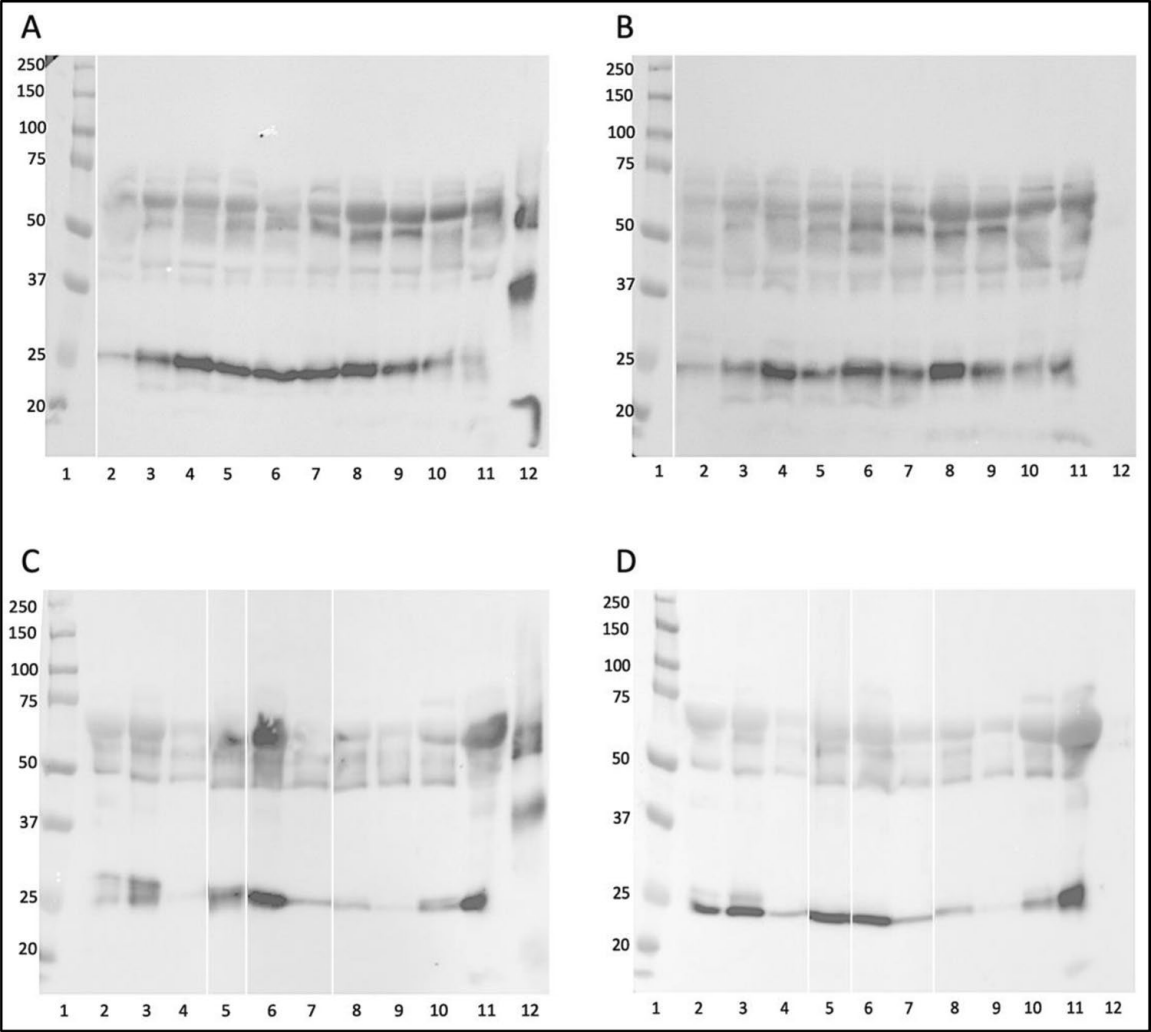
WB analysis of human body fluids with anti-MAGE monoclonal antibody. Ten samples of serum (**A, B**—lane 2–11) and corresponding peritoneal fluid (**C, D**—lane 2–11) from patients with GC were subjected to WB with the anti-MAGE antibody (**A, C**) or as a control with secondary antibody anti-mouse IgE-HRP (**B, D**). Molecular marker indicated in kDa (lane 1) and MB-mel (lane 12) was run as the controls. The original blots are presented in Supplementary Fig. S7.

The presence of MAGE antigen was next verified in body fluid samples from bigger number of subjects. Our previous observation suggested that the sections from different cancer tissues show positive reactivity with the anti-MAGE antibody (manuscript in preparation). The peritoneal fluid is often collected during the diagnostic laparoscopy. It presents a good target specimen for identification of the cancer markers released from the growing tumor and it is used for determination of some diagnostic markers^50^. Therefore, inhere we aimed to study the presence of MAGE antigen in body fluids—blood and peritoneum of the GC patients. For this purpose, WB analysis with the anti-MAGE antibody was performed on 10 serum samples (Fig. 4). Several bands were marked in patients’ serum, including proteins around 60 kDa and 25 kDa (Fig. 4A, lane 2–11), suggesting a reactivity with MAGE antigen, similar like for MB-mel used as a control (Fig. 4A, lane 12). At the same time, the secondary anti-mouse IgE-HRP antibody staining was weaker for some samples (lane 3, 4, 5, 7, 9, 10, Fig. 4B) that suggests the specific binding of the monoclonal anti-MAGE antibody to the serum proteins from patients with GC.

Further, we tested the samples of peritoneal fluids that have been previously collected along the blood from the same patients with GC. The samples transferred on the membrane were exposed to the anti-MAGE monoclonal antibody (Fig. 4C) and interestingly they also showed presence of MAGE antigen on some proteins of mass about 60 kDa. The control staining with the secondary anti-mouse IgE-HRP antibody showed weaker staining for the corresponding bands (lane 2, 3, 5, 6, 7, 8, 10, 11, Fig. 4D), suggesting that MAGE antigen in addition to blood is also present on some proteins in peritoneal fluids of GC patients.

## Discussion

Antibodies can be used as powerful tools to identify trace amount of an analyte in a sample. The antigen-binding properties determine both the sensitivity and selectivity of the immunoassay. The high specificity of antibody is fundamental to their natural function^51^. The generated earlier anti-MAGE antibody specifically binds MAGE antigen synthesized on various carrier proteins (Supplementary Fig. S1). We have previously reported no cross-reactivity of the anti-MAGE antibody with other known AGEs^4^. The specificity was also confirmed in competitive ELISA, with model MAGE generated on MB and protein-fee lysine (Supplementary Fig. S2). Using the anti-MAGE monoclonal antibody, we have identified some proteins (~ 25 and 75 kDa) in human blood from the healthy donor that were clearly separated on a 2D electrophoresis and contained MAGE antigen (Fig. 1). This encouraged us to attempt extraction of the in vivo MAGE antigen from human blood. MAGE was isolated from the diabetic serum (pooled from several patients) and from the donors’ plasma (healthy individuals). The extracted proteins were analyzed by WB (Fig. 2B) and mass spectrometry showing the presence of 59.4 kDa (1S) and 50.0 kDa (2S) proteins in the blood of diabetic patients in contrast to 61.7 kDa (1P) and 49.3 kDa (2P) proteins in the blood of healthy donors. In both samples the proteins with mass over 25 kDa were identified. We suspect these are the light chains of the secreted immunoglobulins^52^ however it needs further confirmation.

In the next step, several types of antibodies specific for different proteins and mass spectrometry analysis were employed for identity verification of the proteins (extracted from human blood) modified with MAGE antigen. In sample 1S (59.4 kDa) the human serum albumin (HSA) showed the best score and 38.8% sequence coverage. This protein was confirmed by WB with the specific anti-HSA antibody (Fig. 3A). Albumin is the most abundant plasma protein and accounts for over 50% of serum proteins in healthy subjects. It plays a key role in maintaining oncotic pressure and is known to be a versatile protein carrier for the transport of various endogenous and exogenous ligands^53^. HSA has a half-life of approximate 3 weeks, sufficient for significant glycation progressing to AGE formation^54^. The structure of HSA consists of a single polypeptide chain of 585 amino acids with a theoretical molecular mass of 66.438 kDa^55^. The molecular mass calculated from spectrometric analysis shows 71.317 kDa (Table 1) and the difference might result from the protein in vivo modifications^56^. It has been proven that this protein is subject to several chemical modifications, including acetylation, oxidation, glycosylation, glycation, carbonylation, and phosphorylation affecting its binding and antigenic properties^57^. After sample separation by SDS-PAGE, the protein molecular mass was calculated to be 59.4 kDa (Fig. 3). Migration of proteins in a polyacrylamide gel in the presence of SDS, which does not correlate with the molecular mass formula, is called “gel shift” and appears to be fairly common but has not yet been fully clarified^58^. This effect is explained by differences in the secondary and tertiary structure of the protein, the intrinsic net charge of the protein, and the amount of bound SDS^58–61^. The literature shows that post-translational modifications of albumin cause changes in the secondary and tertiary structure of HSA, causing e.g. reduced content of α-helices in the structure of polypeptide^62^. It may affect the degree of detergent loading and result in a difference between the actual mass of the protein and the mass calculated from the electrophoretic migration in a gel. The difference in theoretical and observed molecular mass however does not challenge the identity of HSA as the in vivo MAGE-carrier, since it has been confirmed by mass spectrometry (Table 2).

In the sample 1S (Fig. 2B; Table 1), apart from albumin, the α-1-antitrypsin and heavy chains of IgA and IgG were also identified with high scores. Water-soluble and tissue-diffusing α-1-antitrypsin is a circulating glycoprotein with a molecular mass of 52 kDa^63^. Its primary function is to protect tissues from enzymes, such as neutrophil elastase and proteinase 3, released during inflammation^64^. The half-life in the blood of α-1-antitrypsin is 4–5 days, thus is not sufficient for generation of AGEs, and only allows the formation of Amadori products^54,63^. However, the presence of other MAGE-analogous structures on this protein that result from other modifications cannot be ruled out, especially that the origin of the in vivo MAGE has not been elucidated so far. The presence of immunoglobulins G and A in the 1S sample was also excluded by the WB analysis (Fig. 3; Table 2), based on the reaction with specific antibodies showing bands of different molecular mass in comparison to the result of WB with the anti-MAGE antibodies (Fig. 2B).

The sample 1P corresponding to the proteins with a mass of about 61.7 kDa (Fig. 3B) showed presence of fibrinogen α (highest score) and IgA heavy chain (highest sequence coverage) (Table 2). Fibrinogen is a multifunctional acute-phase plasma protein, playing a key role in hemostasis and the coagulation cascade. It occurs in the blood plasma of healthy people at concentrations of 1.5–4 mg/ml. This protein consists of two identical subunits containing three polypeptide chains referred to as Aα, Bβ, and γ chains with a total mass of approximately 340 kDa^65^. According to the literature, the molecular mass of the fibrinogen α chain is ~ 95 kDa^66^, which significantly exceeds the value of the mass determined by SDS-PAGE and WB (Fig. 3). Moreover, fibrinogen has a short blood half-life of 3–5 days^67^ and its glycation seems unlikely. Thus it was not considered in our further studies, but other MAGE analogous structures (different in vivo origin) might mediate binding to the anti-MAGE antibody. The mass spectrometry (Table 2) and immunoblotting analysis provided evidence that sample 1P consists of immunoglobulins (Fig. 3D). The protein band with a mass of 61.5 kDa, evidenced with the anti-IgA antibody, correlated with the mass of the band evidenced with the anti-MAGE antibody (Fig. 3B, band 1P). Theoretical mass of the IgA heavy chain constant region (38.49 kDa) reflects the specific peptide identified by mass spectrometry without considering the variable domain of the whole heavy chain molecule. In electrophoresis, we observed the entire heavy chain of IgA migrating around 60 kDa as it is found in literature^68,69^. Immunoglobulin A is the second most abundant antibody in human blood. It neutralizes toxins and prevents from microbial invasion through the mucosa epithelial barrier. Although this protein has a fairly short half-life of 5–7 days ^70^, there are disease states in which the elimination of IgA is impaired and the protein is deposited in the tissues^71^, allowing for its glycation.

In the sample 2S (serum of diabetic patients) and in the sample 2P (plasma of healthy donors) heavy chain IgG1 (sequence coverage 30 and 28.7%, respectively) was identified by mass spectrometry as the protein with the highest identification score (Table 2). The theoretical molecular mass of this protein is 49.93 kDa and it is consistent with the protein band observed in WB with anti-MAGE antibody (50.0 and 49.3 kDa for 2S and 2P bands, respectively) (Fig. 3, Table 2). In addition, the same size proteins were revealed upon membrane staining with anti-IgG antibody (50.0 kDa for band 7 and 49.2 kDa for band 8 (Fig. 3C, Table 2). Literature describes the human IgG heavy chain as a protein of about 50 kDa^72^, which is in agreement with our observation in WB for both the positive control and test samples (Fig. 3). Slightly lower mass of IgG in the sample 2P (healthy donor) may indicate a lower degree of modification of this protein compared to the IgG in diabetic patient sera. Constant regions of IgG3 and IgG2 heavy chains were also identified in sample 2S, and constant regions of IgG2 and IgG4 heavy chains in sample 2P. The antibodies used in immunoblotting to verify the presence of IgG in the sample are directed to the Fc region of human IgG, which did not allow for the precise determination of the subclass of the identified protein. IgG accounts for about 75% of human serum immunoglobulins and is the primary antibody of the secondary immune response. It has a half-life of 23 days, which is sufficient to generate AGEs through the glycation process^73^.

In the same sample 2P the other proteins, i.e. β and γ chains of fibrinogen have been identified (Table 2) that theoretical mass was calculated as 56.58 kDa and 52.11 kDa. In the studies by Repetto et al*.* fibrinogen β chain migrated at about 50, 40 and 37 kDa^74^, while in the studies of Luo et al*.* the mass of the β chain was determined at 52 kDa and the γ chain at 46 kDa^75^. The observed masses only slightly differ from the mass of the band determined by us (49.3 kDa, Fig. 3B) on a membrane probed with the anti-MAGE antibody. The short half-life of this protein makes it less attractive target for glycation, therefore we focused on IgG identified with a higher score.

Our data suggest that MAGE antigen is present in vivo on several proteins, mainly albumin, IgG and IgA—the main components of blood plasma, accounting for over 70% of all plasma proteins (mainly HSA and IgG)^54^. This is the first report showing specific proteins modified with MAGE found in serum of the diabetic patients (albumin and IgG) and in plasma of the healthy people (IgG and IgA). Results suggest that the MAGE antigen is physiologically present on immunoglobulins for which glycation is a common post-translational modification^76^, but in pathology and oxidative stress formation of MAGE antigen can also occur on other proteins, like albumin. These observations require further confirmation in a larger sample cohort. We assume that IgA modified with MAGE identified in the plasma sample from the healthy donor and not isolated from the serum sample of the diabetic patients, may be explained by abundance of MAGE-modified albumin and IgG under hyperglycemic conditions that saturated the Sepharose-anti-MAGE resin, preventing from binding the proteins of a lower abundance. Further studies to identify additional MAGE-modified proteins in human blood should consider the changes in the immunoprecipitation protocol, i.e. higher concentration of the anti-MAGE antibody and sample re-precipitation.

Significance of our study refers to the glycation-induced change in function of the circulating natural antibodies and potentially some therapeutic antibodies used recently more often in clinics. AGEs increase immunogenicity of immunoglobulins and cause production of antibodies against this modified protein^77^, as reported in type 2 diabetes^78^, atherosclerosis^79^, and rheumatoid arthritis (RA)^80–82^. Glycation is also a suspected cause of decreased elimination of IgA by the liver and the protein accumulation in blood^83^. Serum IgA levels are significantly higher in diabetic patients than in healthy subjects^84–86^. High levels of IgA is a sign of diabetic nephropathy^86^, the most commonly recognized type of glomerular disease in the world and manifested by the formation of mesangial IgA deposits^87^. Glycation of HSA have also serious impact on its function in defense against oxidative stress^88^, transport of various ligands, and regulating of an oncotic pressure due to changes in the secondary and tertiary structure^89^. HSA glycation status is associated with development of autoantibodies in diabetic patients^90,91^ and progression of cardiovascular disease^92^. In this paper we present for the first time that several MAGE-modified proteins are present not only in blood but also in a peritoneal fluid collected from the GC patients (Fig. 4). Similarly, Deng et al. reported an accumulation of the glucose-derived AGEs in tumor and blood of the GC patients^49^, suggesting a potential role of MAGE in cancer. Therefore, some proteins with molecular mass about 25 kDa and 60 kDa modified by MAGE found in our study in peritoneal fluid (suggesting immunoglobulin protein and HSA) will be further investigated to determine the protein identity and relevance to disease pathology and progression. The interesting direction will be also further studies on plasma from diabetic patients to verify the presence of MAGE on fibrinogen in this pathological state.

In summary, the presented data add to the knowledge on protein modifications in vivo and their potential role in diabetes, cancer, and adaptive immune response associated with modification of the circulating antibodies.

## Methods

All chemicals were from Sigma-Aldrich (Saint Louis, MO, USA), unless otherwise stated. All methods were performed in accordance with the relevant guidelines and regulations.

### Human body fluids

In this study we have used plasma or serum collected from the healthy donors (Local Bioethics Committee at Medical University of Wroclaw, KB-770/2018 and Local Bioethics Committee at Medical University of Lublin, KE-0254/157/2016) and serum from the patients with diabetes (Local Bioethics Committee at Medical University of Wroclaw, KB-384/2012, donated by dr Agnieszka Bronowicka-Szydełko). Additionally, we used serum and the corresponding peritoneal fluid collected from the patients with GC described in our earlier studies^50^ (Local Bioethics Committee at Medical University of Lublin, KE–0254/182/2018). The study was approved by the indicated ethics committee and the informed consent was obtained from all subjects.

### MAGE antigen synthesis

The high molecular mass glycation products (HMW-MAGE) used in this study were generated in dry conditions (MWG) as described earlier^4^. Briefly, the miliQ water solution mixtures of melibiose (mel) and one of a model proteins: horse myoglobin (MB), bovine serum albumin (BSA) or rabbit immunoglobulin G (rIgG), in 100:1 molar ratio (carbohydrate:protein) were prepared and subjected to MWG reaction. Next, the samples were dissolved in milliQ water and centrifuged at 5000 × g, 15 min to remove any insoluble precipitates. The obtained supernatants were washed several times with milliQ water on Amicon Ultra-15 centrifuge filters (Merck Millipore, Burlington, MA, USA) with a 30 kDa cut-off to remove the unreacted substrates. The resulting glycated proteins were frozen at − 80 °C, lyophilized, and stored at − 20 °C until further studies.

### SDS-PAGE and Western blotting

The model protein and samples immunoprecipitated with the Sepharose-anti-MAGE resin were diluted 1:1 with Non-Reducing Lane Marker Sample Buffer (Thermo Scientific, Waltham, MA, USA), supplemented with 100 mM β-mercaptoethanol and denatured by incubation at 100 °C for 5 min. The samples were separated on 12% polyacrylamide gel using a Mini-Protean Tetra Cell apparatus (Bio-Rad, Hercules, CA, USA) for 90 min under constant voltage of 100 V. The gel was next stained with Quick Coomassie Protein Stain (ProteinArk, Sheffield, UK). The results were visualized on a PXi Touch gels and blots detection and analysis system (SYNGENE, Cambridge, UK). The molecular mass of the selected bands was determined with LabImage gel analysis software (Kapelan Bio-Imaging, Leipzig, Germany). For WB the proteins separated on a polyacrylamide gel were next transferred to a PVDF membrane (0.45 μm, Immobilon-P Transfer Membrane PVDF, Merck-Millipore, Burlington, MA, USA) using a Mini Trans-Blot Cell apparatus (Bio-Rad, Hercules, CA, USA). The transfer was carried out for 1 h at constant voltage of 100 V (for model protein). Then the membrane was blocked with 5% solution of skimmed milk powder (SM Gostyń, Poland) in TBS-T (Tris with 0.05% Tween 20) for 1 h, at room temp or overnight at 4 °C. After washing 3 times with TBS-T, the membrane was incubated for 2 h, at room temp with one of the antibodies: goat anti-human IgG-HRP (Abcam, Cambridge, UK), goat anti-human IgA-HRP (Abcam, Cambridge, UK) diluted 1:15 000 in phosphate-buffered saline (PBS) pH 7.4 (single step, no secondary antibodies), rabbit anti-HSA (human serum albumin, Thermo Scientific, Waltham, MA, USA) diluted 1:5 000 or with culture medium from the hybridoma cells producing mouse anti-MAGE monoclonal antibodies, with incubation carried overnight at 4 °C, followed by 3 times washing with TBS-T solution. Next, when appropriate, the membranes were incubated for 1.5 h, at room temp with anti-rabbit IgG-HRP antibody (Abcam, Cambridge, UK) diluted 1:2 000 or goat anti-mouse IgE-HRP antibody (Origene, Rockville, MD, USA) diluted 1:5 000 in PBS. After washing 5 times with TBS-T, antibody binding was visualized by incubation with SuperSignal™ West Pico PLUS Chemiluminescent Substrate (Thermo Scientific, Waltham, MA, USA). The results were photographed using PXi Touch gels and blots detection and analysis system (SYNGENE, Cambridge, UK).

Electrophoresis of the human body fluids (blood and peritoneal fluid) from GC patients was performed on miliQ water-diluted samples (1:1) subjected to protein precipitation with cold methanol (chilled to − 20 °C) by mixing 1 volume of sample with 4 parts of 100% methanol. The protein pellet in each sample obtained after centrifugation (14 000 × g, 5 min, 4 °C) was dissolved in the original sample volume of RIPA buffer (EMD Milipore, Billerica, MA, USA), mixed with a Laemmli sample buffer containing 20% β-mercaptoethanol, and denatured by incubation at 97 °C for 10 min. Equal volume of each sample was separated on 8% polyacrylamide gel at constant voltage of 200 V for 50 min. Next the proteins were transferred onto the PVDF membrane with constant voltage of 200 V (for human body fluids). After incubation with a blocking solution (5% solution of skim milk powder from Carl Roth, Karlsruhe, Germany dissolved in PBS containing 0.05% Tween (PBS-T)) for 1 h at room temp the membrane was incubated overnight at 4 °C with antibodies anti-MAGE diluted in blocking solution. Next, the excess of antibodies was washed out 3 times with PBS-T and the membrane was incubated for 4 h with secondary antibody anti-mouse IgE-HRP (Origene, Rockville, MD, USA) diluted 1:8 000 in PBS-T. After 3 times washing with PBS-T, antibody binding was visualized and photographed using ChemiDoc MP V3 imaging system (BioRad, Hercules, CA, USA) after incubation with a Clarity Western ECL Substrate (BioRad, Hercules, CA, USA).

### 2-D Electrophoresis and identification of the proteins for mass spectrometry analysis

Sample (120 µl) of human serum from the healthy donor, previously diluted 1:1 in miliQ water, was mixed with cold methanol (480 µl) and incubated for 20 min at − 20 °C to precipitate proteins. After centrifugation at 13 000 rpm for 5 min, the pellet was retained and subjected to the sample preparation procedure using the Cleanup solution according to the manufacture protocol (BioRad, Hercules, CA, USA). The sample was finally reconstituted in 150 µl of Rehydration solution (8 M urea, 2% CHAPS, 50 mM DTT, 0,2% (w/v) Bio-Lyte 3/10 ampholytes, trace of Bromophenol Blue). Before application on the gel the sample was vortexed, sonicated for 5 min and centrifuged at 13 000 rpm for 3 min to remove any residual insoluble material. Isoelectric focusing was performed on a strip within pH 3–10 using a BioRad PROTEAN i12 IEF Cell (BioRad, Hercules, CA, USA) with gradient parameters: 250 V linear for 20 min, 4000 V linear for 2 h, rapid 4000–10 000 VHr, hold at 500 V and rehydration for 14 h. Next day the strip was dried on a paper towel and equilibrated in a tray with solution I (6 M Urea, 2% SDS, 0.375 M Tris–HCl pH 8.8, 20% glycerol, 2%, DTT) and solution II (6 M Urea, 2% SDS, 0.375 M Tris–HCl pH 8.8, 20% glycerol, iodoacetamide) for 10 min each. The second-dimension electrophoresis was carried on 8% polyacrylamide gel at 200 V for 42 min. Next the gel was rinsed 3 × for 10 min with miliQ water and stained with Bio-Safe Coomassie Stain (BioRad, Hercules, CA, USA) or transferred to the PVDF membrane at 150 V for 90 min. The membrane was subjected to WB with anti-MAGE mAb diluted in bocking solution (as above for peritoneal fluids WB) followed by anti-mouse IgE-HRP (Origene Rockville, MD, USA) diluted 1:6 000 in PBS-T. The membrane serving as the negative control was incubated only with secondary antibody without the anti-MAGE antibody exposure. The results were photographed using an ChemiDoc MP V3 imaging system (BioRad, Hercules, CA, USA) after incubation with a Clarity Western ECL Substrate (BioRad, Hercules, CA, USA).

The protein spots visualized on the gel stained with Coomassie were photographed and manually correlated using the PhotoShop software with the spots of proteins reacting with the anti-MAGE mAb on the PVDF membrane and were excised from the gel. For mass spectrometry analysis the proteins that did not show reactivity with the anti-mouse-IgE-HRP antibody (negative control) were selected. The excised gel pieces were stored at − 20 °C until analysis.

### Isolation of MAGE-modified proteins from human blood

The analogue of MAGE present naturally in human blood was extracted from the samples collected in the Military Center for Blood Donation and Haemotherapy in Wroclaw (honorary blood donors) and the Department of Angiology, Diabetology and Hypertension in Wroclaw (diabetic patients). The presence of the MAGE antigen in the tested diabetic sera was previously confirmed by competitive ELISA^5^. MAGE extraction was performed using the purified murine anti-MAGE monoclonal antibody (described in Supplementary data) immobilized on Sepharose resin using the Pierce Direct IP Kit (Thermo Scientific, Waltham, MA, USA). The procedure was carried out in accordance with the manufacturer's instruction. Briefly, for each sample, 8 µg of anti-MAGE antibody was mixed with 20 µl of Sepharose resin suspension and incubated for 2 h, at room temp on a shaker (BioSan, Riga, Latvia) at low speed. The Sepharose-anti-MAGE resin was next washed and incubated with serum sample (mixed from several patients) or blood plasma containing 1 mg of protein (for 2 h, at room temp and then overnight at 4 °C with gentle rocking), followed by incubation with the blocking solution (1 M Tris–HCl). The resin incubated only with a blocking solution, without anti-MAGE antibody was used as the negative control. After the resin was appropriately washed, the resin-bound material was eluted with the elution solution (25–75 µl). The collected samples were stored at − 20 °C until further analysis.

### Identification of MAGE-modified proteins present in human blood by mass spectrometry

Proteins extracted from human blood on Sepharose-anti-MAGE resin were separated on a polyacrylamide gel (SDS-PAGE) and stained. The pieces of gel with protein bands and the spots from 2-D gel (corresponding to the proteins identified in WB with anti-MAGE Ab) were excised and after drying, the proteins were reduced with 10 mM DTT (dithiothreitol), alkylated with 50 mM iodoacetamide, and digested with trypsin at 37 °C overnight. The obtained peptides were extracted from the gel with 0.1% TFA (trifluoroacetic acid) containing 2% acetonitrile. Mass spectrometry analysis was performed at the Environmental Mass Spectrometry Laboratory, Institute of Biochemistry and Biophysics, Polish Academy of Sciences in Warsaw. The analysis employed a liquid chromatography coupled with an Orbitrap mass spectrometry (Thermo Scientific, Waltham, MA, USA). Protein identification was performed using the MASCOT software (Matrix Science, Boston, MA, USA) by comparison with the UniProt protein sequence database.

## Supplementary Information

Supplementary Information.

## References

[1] Pietkiewicz J, Seweryn E, Bartys̈ A, Gamian A (2008). Receptory końcowych produktów zaawansowanej glikacji-znaczenie fizjologiczne i kliniczne. Postepy Higieny i Medycyny Doswiadczalnej.

[2] Nagai R (2016). Antibody-based detection of advanced glycation end-products: Promises vs limitations. Glycoconj. J..

[3] Ashraf JM (2015). Recent advances in detection of AGEs: Immunochemical, bioanalytical and biochemical approaches. IUBMB Life.

[4] Staniszewska M (2021). The melibiose-derived glycation product mimics a unique epitope present in human and animal tissues. Sci. Rep..

[5] Bronowicka-szydełko A, Krzystek-korpacka M, Gacka M (2021). Association of novel advanced glycation end-product ( AGE10) with complications of diabetes as measured by enzyme-linked immunosorbent assay. J. Clin. Med..

[6] Indyk D, Bronowicka-Szydełko A, Gamian A, Kuzan A (2021). Advanced glycation end products and their receptors in serum of patients with type 2 diabetes. Sci. Rep..

[7] Czech M (2021). The genotoxic and pro-apoptotic activities of advanced glycation end-products (MAGE) measured with micronuclei assay are inhibited by their low molecular mass counterparts. Genes.

[8] O’Connell KJ (2013). Metabolism of four α-glycosidic linkage-containing oligosaccharides by Bifidobacterium breve UCC2003. Appl. Environ. Microbiol..

[9] O’Callaghan A, van Sinderen D (2016). Bifidobacteria and their role as members of the human gut microbiota. Front. Microbiol..

[10] Yoon MY, Hwang HJ (2008). Reduction of soybean oligosaccharides and properties of α-d-galactosidase from Lactobacillus curvatus R08 and Leuconostoc mesenteriodes JK55. Food Microbiol..

[11] Baú TR, Garcia S, Ida EI (2015). Changes in soymilk during fermentation with kefir culture: Oligosaccharides hydrolysis and isoflavone aglycone production. Int. J. Food Sci. Nutr..

[12] Schievano E, Tonoli M, Rastrelli F (2017). NMR quantification of carbohydrates in complex mixtures: A challenge on honey. Anal. Chem..

[13] Mao B (2018). In vitro fermentation of raffinose by the human gut bacteria. Food Funct..

[14] Sandek A (2012). Studies on bacterial endotoxin and intestinal absorption function in patients with chronic heart failure. Int. J. Cardiol..

[15] Fan X, Monnier VM (2021). Protein posttranslational modification (PTM) by glycation: Role in lens aging and age-related cataractogenesis. Exp. Eye Res..

[16] Ahmad S (2013). Inhibitory effect of metformin and pyridoxamine in the formation of early, intermediate and advanced glycation end-products. PLoS ONE.

[17] Rehman S, Faisal M, Alatar AA, Ahmad S (2020). Physico-chemical changes induced in the serum proteins immunoglobulin g and fibrinogen mediated by methylglyoxal. Curr. Protein Pept. Sci..

[18] DeGroot J (2004). Accumulation of advanced glycation end products as a molecular mechanism for aging as a risk factor in osteoarthritis. Arth. Rheum..

[19] Fournet M, Bonté F, Desmoulière A (2018). Glycation damage: A possible hub for major pathophysiological disorders and aging. Aging Dis..

[20] Akhter F, Khan MS, Alatar AA, Faisal M, Ahmad S (2016). Antigenic role of the adaptive immune response to d-ribose glycated LDL in diabetes, atherosclerosis and diabetes atherosclerotic patients. Life Sci.

[21] Akhter F, Salman Khan M, Faisal M, Alatar AA, Ahmad S (2017). Detection of circulating auto-antibodies against ribosylated-LDL in diabetes patients. J. Clin. Lab. Anal..

[22] Alavi P, Yousefi R, Amirghofran S, Karbalaei-Heidari HR, Moosavi-Movahedi AA (2013). Structural analysis and aggregation propensity of reduced and nonreduced glycated insulin adducts. Appl. Biochem. Biotechnol..

[23] Miele C (2003). Human glycated albumin affects glucose metabolism in l6 skeletal muscle cells by impairing insulin-induced insulin receptor substrate (IRS) Signaling through a protein Kinase Cα-mediated mechanism. J. Biol. Chem..

[24] Guo Q (2009). Methylglyoxal contributes to the development of insulin resistance and salt sensitivity in Sprague-Dawley rats. J. Hypertens..

[25] Lin J-A, Wu C-H, Yen G-C (2018). Perspective of advanced glycation end products on human health. J. Agric. Food Chem..

[26] Younis N (2008). Glycation as an atherogenic modification of LDL. Curr. Opin. Lipidol..

[27] Gonen B, Baenziger J, Schonfeld G, Jacobson D, Farrar P (1981). Nonenzymatic glycosylation of low density lipoproteins in vitro: Effects Cell-Interactive properties. Diabetes.

[28] Yamagishi S, Ueda S, Matsui T, Nakamura K, Okuda S (2008). Role of ADVANCED GLYCATION END PRODUCTS (AGEs) and oxidative stress in diabetic retinopathy. Curr. Pharm. Des..

[29] Yamagishi SI, Matsui T (2010). Advanced glycation end products, oxidative stress and diabetic nephropathy. Oxid. Med. Cell. Longev..

[30] Aronson D (2003). Cross-linking of glycated collagen in the pathogenesis of arterial and myocardial stiffening of aging and diabetes. J. Hypertens..

[31] Bell DSH (2003). Heart failure: The frequent, forgotten, and often fatal complication of diabetes. Diab. Care.

[32] Perrone A, Giovino A, Benny J, Martinelli F (2020). Advanced glycation end products (AGEs): Biochemistry, signaling, analytical methods, and epigenetic effects. Oxidat. Med. Cell. Longev..

[33] Sorci G, Riuzzi F, Giambanco I, Donato R (2013). RAGE in tissue homeostasis, repair and regeneration. Biochim. Biophys. Acta Mol. Cell Res..

[34] Schmidt AM (2017). RAGE and Implications for the pathogenesis and treatment of cardiometabolic disorders: Spotlight on the Macrophage. Arterioscler. Thromb. Vasc. Biol..

[35] Ramasamy R, Yan SF, Schmidt AM (2012). The diverse ligand repertoire of the receptor for advanced glycation endproducts and pathways to the complications of diabetes. Vascul. Pharmacol..

[36] Liu, T., Zhang, L., Joo, D. & Sun, S.-C. NF-κB signaling in inflammation. (2017) 10.1038/sigtrans.2017.23

[37] Ott C (2014). Role of advanced glycation end products in cellular signaling. Redox Biol..

[38] Dariya B, Nagaraju GP (2020). Advanced glycation end products in diabetes, cancer and phytochemical therapy. Drug Discov. Today.

[39] Lin JA, Wu CH, Lu CC, Hsia SM, Yen GC (2016). Glycative stress from advanced glycation end products (AGEs) and dicarbonyls: An emerging biological factor in cancer onset and progression. Mol. Nutr. Food Res..

[40] Liang H (2020). Advanced glycation end products induce proliferation, invasion and epithelial-mesenchymal transition of human SW480 colon cancer cells through the PI3K/AKT signaling pathway. Oncol. Lett..

[41] Bao JM (2015). AGE/RAGE/Akt pathway contributes to prostate cancer cell proliferation by promoting Rb phosphorylation and degradation. Am. J. Cancer Res..

[42] Ahmad S (2018). Oxidation, glycation and glycoxidation: The vicious cycle and lung cancer. Semin. Cancer Biol..

[43] Chen H (2017). Advanced glycation end products promote ChREBP expression and cell proliferation in liver cancer cells by increasing reactive oxygen species. Medicine.

[44] Jabir NR, Ahmad S, Tabrez S (2018). An insight on the association of glycation with hepatocellular carcinoma. Semin. Cancer Biol..

[45] Omofuma OO (2020). Dietary advanced glycation end-products (AGE) and risk of breast cancer in the prostate, lung, colorectal and ovarian cancer screening trial (PLCO). Cancer Prev. Res..

[46] Wang X, Zhang R, Zhang L, Tian Z (2019). Glycated serum proteins: High in pancreatic cancer and low in preeclampsia. Prog. Mol. Biol. Transl. Sci..

[47] Shahab U (2018). The receptor for advanced glycation end products: A fuel to pancreatic cancer. Semin. Cancer Biol..

[48] Abe R (2004). Regulation of human melanoma growth and metastasis by AGE-AGE receptor interactions. J. Inv. Dermatol..

[49] Deng R (2017). Glucose-derived AGEs enhance human gastric cancer metastasis through RAGE/ERK/Sp1/MMP2 cascade. Oncotarget.

[50] Gęca K (2022). Kynurenine and anthranilic acid in the peritoneum correlate with the stage of gastric cancer disease Int. J. Tryptophan Res..

[51] Hnasko RM (2015). The biochemical properties of antibodies and their fragments. Methods Mol. Biol..

[52] Schroeder HW, Cavacini L (2010). Structure and function of immunoglobulins. J. Allergy Clin. Immunol..

[53] Raoufinia R (2016). Overview of albumin and its purification methods. Adv. Pharm. Bull..

[54] Vetter SW (2015). Glycated Serum Albumin and AGE Receptors. Advances in Clinical Chemistry.

[55] More J, Bulmer M (2012). Human Serum Albumin: A Multifunctional Plasma Protein. Production of Plasma Proteins for Therapeutic Use.

[56] Schuhmacher M, Glocker MO, Wunderlin M, Przybylski M (1996). Direct isolation of proteins from sodium dodecyl sulfate-polyacrylamide gel electrophoresis and analysis by electrospray-ionization mass spectrometry. Electrophoresis.

[57] Fanali G (2012). Human serum albumin: From bench to bedside. Mol. Aspects Med..

[58] Rath A, Glibowicka M, Nadeau VG, Chen G, Deber CM (2008). Detergent binding explains anomalous SDS-PAGE migration of membrane proteins. PNAS.

[59] Pitt-Rivers R, Impiombato FS (1968). The binding of sodium dodecyl sulphate to various proteins. Biochem. J..

[60] Dunker AK, Kenyon AJ (1976). Mobility of sodium dodecyl sulphate: Protein complexes. Biochem. J..

[61] Shi Y (2012). Abnormal SDS-PAGE migration of cytosolic proteins can identify domains and mechanisms that control surfactant binding. Protein Sci..

[62] Neelofar KM, Ahmad J, Arif Z, Alam K (2016). Elucidating the impact of glucosylation on human serum albumin: A multi-technique approach. Int. J. Biol. Macromol..

[63] de Serres F, Blanco I (2014). Role of alpha-1 antitrypsin in human health and disease. J. Intern. Med..

[64] Lechowicz U, Rudzinski S, Jezela-Stanek A, Janciauskiene S, Chorostowska-Wynimko J (2020). Post-translational modifications of circulating alpha-1-antitrypsin protein. Int. J. Mol. Sci..

[65] Perween S, Abidi M, Faizy AF, Moinuddin (2019). Post-translational modifications on glycated plasma fibrinogen: A physicochemical insight. Int. J. Biol. Macromol..

[66] Fu Y, Grieninger G (1994). Fib420: A normal human variant of fibrinogen with two extended α chains. Proc. Natl. Acad. Sci. USA.

[67] Vilar R, Fish RJ, Casini A, Neerman-Arbez M (2020). Fibrin(ogen) in human disease: Both friend and foe. Haematologica.

[68] Kerr MA (1990). The structure and function of human IgA. Biochem. J..

[69] Suzuki T (2015). Relationship of the quaternary structure of human secretory IgA to neutralization of influenza virus. Proc. Natl. Acad. Sci. USA.

[70] Tomasz Kowalik, J. S. Salivary secretory IgA and its impact on dental caries. *Nowa Stomatologia* 211–214 (2013).

[71] Perše M, Večeri večeri c-Haler Ž (2019). The role of IgA in the pathogenesis of IgA nephropathy. Int. J. Mol. Sci..

[72] Charles A Janeway, J., Travers, P., Walport, M. & Shlomchik, M. J. The structure of a typical antibody molecule. in *Immunobiology: The Immune System in Health and Disease. 5th edition.* (Garland Science, 2001).

[73] NapiórkowsKa-Baran K (2019). Oznaczanie przeciwciał w codziennej praktyce. Część I-właściwości przeciwciał Determination of antibodies in everyday practice: Part I-properties of antibodies. Alergia Astma Immunol..

[74] Repetto O (2018). Quantitative proteomic approach targeted to fibrinogen β chain in tissue gastric carcinoma. Int. J. Mole. Sci..

[75] Luo Y, Shi J, Li J (2015). Peroxynitrite induced fibrinogen site identification. Bio-Med. Mater. Eng..

[76] Wei B, Berning K, Quan C, Zhang YT (2017). Glycation of antibodies: Modification, methods and potential effects on biological functions. MAbs.

[77] Ahmad S, Moinuddin, Ali A (2012). Immunological studies on glycated human IgG. Life Sci..

[78] Islam S (2017). Neo-epitopes generated on hydroxyl radical modified glycatedigg have role in immunopathology of diabetes type 2. PLoS ONE.

[79] Korça E, Piskovatska V, Börgermann J, Navarrete Santos A, Simm A (2020). Circulating antibodies against age-modified proteins in patients with coronary atherosclerosis. Sci. Reports.

[80] Newkirk MM (2003). Advanced glycation end-product (AGE)-damaged IgG and IgM autoantibodies to IgG-AGE in patients with early synovitis. Arth. Res. Ther..

[81] Tai AWH, Newkirk MM (2000). An autoantibody targeting glycated IgG is associated with elevated serum immune complexes in rheumatoid arthritis (RA). Clin. Exp. Immunol..

[82] Ryan BJ, Nissim A, Winyard PG (2014). Oxidative post-translational modifications and their involvement in the pathogenesis of autoimmune diseases. Redox Biol..

[83] Kawata Y (2021). Detailed structure and pathophysiological roles of the IgA-albumin complex in multiple myeloma. Int. J. Mol. Sci..

[84] Kawasaki I (1998). Renal dysfunction worsened by superimposition of IgA glomerulonephritis in a patient with overt diabetic nephropathy [6]. Nephron.

[85] Nasr SH, Share DS, Vargas MT, D’Agati VD, Markowitz GS (2007). Acute poststaphylococcal glomerulonephritis superimposed on diabetic glomerulosclerosis. Kidney Int..

[86] Kanauchi M, Kawano T, Dohi K (2000). Serum IgA levels in patients with diabetic nephropathy and IgA nephropathy superimposed on diabetes mellitus. Diab. Res. Clin. Pract..

[87] Perše M, Večeri-Večeric-Haler Ž (2019). The role of IgA in the Pathogenesis of IgA nephropathy. Int. J. Mol. Sci..

[88] Matsuda R, Anguizola J, Joseph KS, Hage DS (2011). High-performance affinity chromatography and the analysis of drug interactions with modified proteins: Binding of gliclazide with glycated human serum albumin. Anal. Bioanal. Chem..

[89] Mendez DL, Jensen RA, McElroy LA, Pena JM, Esquerra RM (2005). The effect of non-enzymatic glycation on the unfolding of human serum albumin. Arch. Biochem. Biophys..

[90] Arif B (2012). Structural and immunological characterization of Amadori-rich human serum albumin: Role in diabetes mellitus. Arch. Biochem. Biophys..

[91] Neelofar K, Ahmad J (2017). An overview of in vitro and in vivo glycation of albumin: A potential disease marker in diabetes mellitus. Glycoconj. J..

[92] Paradela-Dobarro B (2019). Inflammatory effects of in vivo glycated albumin from cardiovascular patients. Biomed. Pharmacother..

