# Supplementary material for: Proteins in human body fluids contain in vivo antigen analog of the melibiose-derived glycation product: MAGE
Source: Sci Rep. 2022 May 7;12:7520. doi: 10.1038/s41598-022-11638-2 (PMC9079080; doi:10.1038/s41598-022-11638-2)
Supplement: Supplementary file 1 — Supplementary Information. [file 41598_2022_11638_MOESM1_ESM.docx]

**Proteins in human body fluids contain *in vivo* antigen analog of the melibiose-derived glycation product - MAGE**

Kinga Gostomska-Pampuch^1,2^, Andrzej Gamian^2^, Karol Rawicz-Pruszyński^3^, Katarzyna Gęca^3^, Joanna Tkaczuk-Włach^4^, Ilona Jonik^5^, Kinga Ożga^5^, Magdalena Staniszewska^5*^

^1^Department of Biochemistry and Immunochemistry, Wroclaw Medical University, Chalubinskiego 10, 50-368 Wroclaw, Poland

^2^Laboratory of Medical Microbiology, Ludwik Hirszfeld Institute of Immunology and Experimental Therapy, Polish Academy of Sciences, Weigla 12, 53-114 Wroclaw, Poland

^3^Department of Surgical Oncology, Medical University of Lublin, Radziwillowska 13, 20-080 Lublin, Poland

^4^Diagnostic Techniques Unit, Collegium Maximum, Medical University of Lublin, Staszica 4/6, 20-081 Lublin, Poland

^5^Centre for Interdisciplinary Research, The John Paul II Catholic University of Lublin, Konstantynów 1J, 20-708 Lublin, Poland

*Correspondence: [magdalena.staniszewska@kul.pl](mailto:magdalena.staniszewska@kul.pl)

**Supplementary Methods**

*Affinity chromatography purification of anti-MAGE antibody*

The HMW-MAGE antigen (MB-mel) at the concentration of 8 mg/ml was immobilized to the activated Sepharose-CL4B resin suspended in 0.1 M bicarbonate buffer, pH 8.3. The suspension was incubated for 24 hours at 4°C on the rotator (Multi Bio RS-24, BioSan, Riga, Latvia). Then, unreacted -CN groups were blocked by incubation with 0.1 M Tris-HCl pH 8.0 for 2 h, at room temperature on the rotator. After incubation, the Sepharose-MAGE resin was washed three times with alternating 0.1 M acetate buffer pH 4.0 and 0.1 M Tris-HCl/0.5 M NaCl pH 8.0. Finally, the resin was washed 4 times with PBS (140 mM NaCl, 2.7 mM KCl, 10 mM Na_2_HPO_4_, 1.8 mM KH_2_PO_4_, pH 7.4), packed in a glass column by gravity and stored at 4°C as a suspension in PBS with the addition of 0.02% sodium azide.

The anti-MAGE monoclonal antibody were purified from the culture medium of the previously obtained hybridoma cells ^1^, cultured in a medium containing 2% of fetal bovine serum (FBS). The medium was applied to the affinity column with immobilized MAGE antigen, with a flow rate of 1 ml/min. After washing the column with a double volume of PBS, the bound antibodies were eluted with 3M KSCN (5 ml). The eluted fractions (1 ml) were monitored for the presence of protein by measuring absorbance at 280 nm with a spectrophotometer (DS-11FX, DeNovix Inc. Wilmington, DE, USA). The pooled protein-containing fractions were concentrated to a final volume of 150 µl on Amicon Ultra-15 type centrifuge filters (Merck Millipore, Burlington, MA, USA) with a cut-off of 30 kDa (5 000 x g, 20 min). The fractions eluted with KSCN were additionally subjected to buffer exchange to PBS on the same filters. The protein concentration in the finally concentrated fractions was determined using the Pierce Coomassie Plus (Bradford) Assay Kit (Thermo Scientific, Waltham, MA, USA), analyzed by SDS-PAGE and ELISA to test for antibody presence and activity. Purified antibody was stored in 50 % glycerol at -20˚C.

*ELISA (Enzyme-Linked Immunosorbent Assay)*

The 96-well plate was incubated overnight at 4°C with a solution of MAGE antigen (0.5 µg /well) in 100 µl of 0.1 M carbonate buffer, pH 9.6. Control wells were coated with the carrier proteins used for the synthesis of glycation products (myoglobin, MB; bovine serum albumin, BSA; rabbit immunoglobulin G, rIgG). Then the plates were washed three times with TBS-T solution (20 mM Tris-HCL, 150 mM NaCl, pH 7.4, 0.05 % Tween-20) and blocked with 5% skimmed milk powder (SM Gostyń, Poland) solution in TBS-T for 1 hour, at room temp. Next, after washing 3 times with TBS-T, 50 µl/well of hybridoma cell culture medium containing anti-MAGE antibody or purified anti-MAGE antibody in a dilution series (100 µl/well) were applied on the ELISA plate. Each dilution point was applied in triplicate and the plate was incubated overnight at 4°C. Then, after washing with TBS-T as before, 100 µl/well of secondary antibodies were applied: goat polyclonal anti-mouse Igs-HRP (Dako, Glostrup, Denmark), reacting with all classes of murine immunoglobulins, diluted 1: 2 000 in PBS or goat anti-mouse- IgE-HRP (Origene, Rockville, MD, USA) diluted 1: 5 000 in PBS. After 2 hours incubation at room temp, an excess of antibodies was washed away with TBS-T and the reaction was induced with 100 µl/well of developing OPD solution containing citrate buffer, pH 4.5 (0.1 M citric acid, 0.1 M sodium citrate), 1.5 mg/ml *ortho*-phenylenediamine (OPD), 0.03% (v/v) H_2_O_2,_ for 10 min in the dark, at room temp. The reaction was stopped with 40% H_2_SO_4_ (50 µl/well) and the obtained color intensity was measured at 490 nm with a Synergy HT spectrophotometer (BioTek Instruments, Winooski, VT, USA). The results were normalized by subtracting the absorbance for a negative control where PBS was applied to a well instead of primary antibody.

*Competitive ELISA*

The 96-well plate was coated with MAGE antigen and blocked as described above. In parallel, a series of dilutions of the tested glycation products (HMW-MAGE) and substrates (MB, melibiose) were prepared in 105 µl of PBS. Solution of the affinity purified anti-MAGE antibody (1.2 mg/ml) diluted 1:125 in PBS was added to 105 µl of sample (final dilution in the well was 1:250). The mixtures were incubated in tubes for 2 h, room temp, and then applied on the plate in duplicates (100 µl/well). The positive control of the assay (100% reaction) was the reactivity of the anti-MAGE antibod at a dilution of 1:250 in PBS, without addition of inhibitors. The plate was incubated for 1.5 h, at room temp, and next overnight at 4°C. After washing the plate three times with TBS-T, the plate was incubated with the secondary antibody and developed reaction was identified as described above. All datapoints were normalized by subtracting the absorbance for a negative control where PBS was applied to a well instead of primary antibodies. The results are given as B/B_0_ ratio, where B indicates the absorbance of the sample with inhibitor and B_0_ the positive control without inhibitor (100% reaction).

LMW-MAGE synthesis

In order to obtain low molecular weight MAGE product (LMW-MAGE) an equimolar mixture (1:1) of the amino acid derivative: Nα-acetyl-L-lysine (NAL) and melibiose (35 mmol/L each) was dissolved in 5 ml milliQ water, frozen at -80°C, and lyophilized. The glycation reaction was performed in a microwave reactor (Initiator, Biotage, Uppsala, Sweden) for 20 min at 60°C and constant power of 200 Watts. After the reaction, the resulting LMW-MAGE was purified by liquid chromatography as previously described ^2^.

**Supplementary Results**

*Anti-MAGE antibody purification and characterization*

The monoclonal anti-MAGE antibody were produced using the previously selected hybridoma cells, determined to be the mouse IgE class ^1^. Supernatant from hybridoma culture was tested with HMW-MAGE antigens: MB-mel, BSA-mel, rIgG-mel (all synthesized under anhydrous conditions, MWG) and in parallel with the unmodified corresponding carrier proteins. The ELISA results confirmed that the generated antibody react specifically with the MAGE products independently of the carrier protein, in the absence of the reactivity with the unmodified proteins (Fig. S1A). Then, the hybridoma cell culture medium was fractionated on 30 ml Sepharose-MAGE column to obtain large scale of pure anti-MAGE antibody. The process was repeated many times, and an exemplary separation profile is illustrated in Fig. S1B. The procedure was optimized and allowed for reproducible collection of pure anti-MAGE antibody (Fig. S1B, KSCN fraction). The potassium thiocyanate-washed material was pooled, dialyzed against PBS, and concentrated on Amicon-type filters to yield a pure anti-MAGE antibody preparation. On average antibody concentration of 1.2 mg/ml in a volume of 45 µl was obtained from 120 ml of the starting medium, that is 45 µg of pure antibodies from 100 ml of hybridoma cell culture medium. The purity of the obtained antibody was tested using the SDS-PAGE method. In the Coomassie stained gel (Fig. S1C) there were 3 bands. The most intense band was observed at a mass level of about 75 kDa, which according to the literature corresponds to the mass of the heavy chain of murine E class immunoglobulin ^3^. There was also a protein band with a mass estimated at approx. 65 kDa and a faintly visible band with a mass of approx. 20 kDa, which corresponds to the mass of the light chain of immunoglobulins, e.g. κ chain ^4^. The antibody protein identity and class were confirmed by mass spectrometry analysis of the 75 kDa band (Fig. S1C). The highest score of 15924 was obtained for the C region (constant) of the immunoglobulin epsilon chain, confirming (sequence coverage 69%) that the sample contains the mouse immunoglobulin class E. The activity of the purified anti-MAGE monoclonal antibody was determined by ELISA on a plate coated with MB-mel MWG in a dilution series (Fig. S1D). Antibody activity was observed at a 0 – 4 000-fold dilution, which corresponds to a concentration of 0.3 µg/ml and is consistent with the expected activity of other monoclonal antibodies.


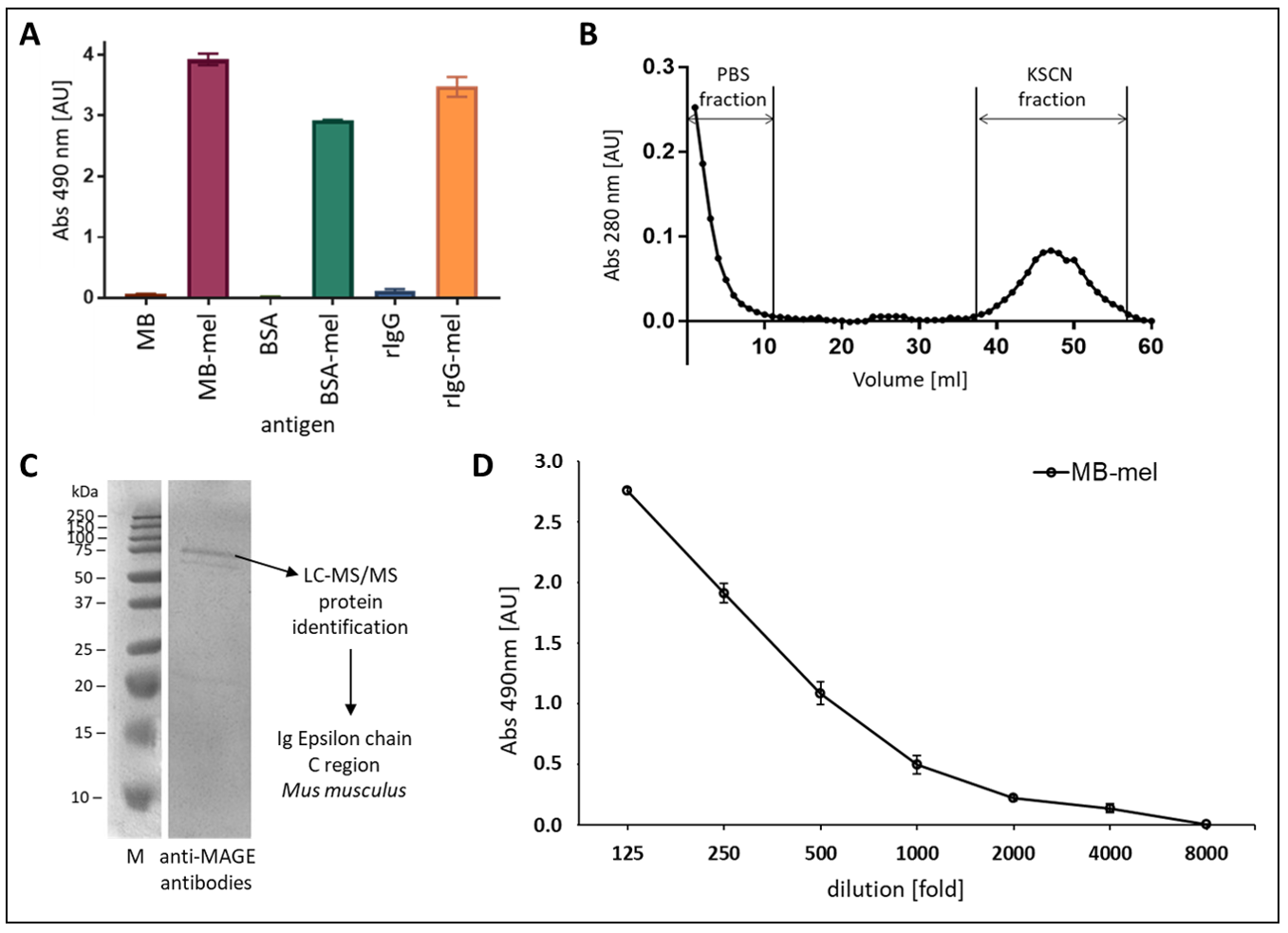


**Fig. S1. Purification and characterization of the anti-MAGE monoclonal antibody.** Reactivity of cell culture medium from the hybridoma cell was tested by ELISA on plate coated with HMW-MAGE antigens: MB-mel, BSA-mel, rIgG-mel and carrier proteins: MB, BSA, rIgG; secondary antibodies: goat anti-mouse Igs-HRP **(A)**. The values show the mean absorbance measured at 490 nm from 2 wells after subtracting the absorbance of the negative sample. Sepharose-MAGE exemplary elution profile of the anti-MAGE antibody **(B)**. The PBS fraction corresponds to the unbound components of the culture medium and the KSCN fraction contains anti-MAGE antibody; eluted material was monitored at 280 nm. The purity of the anti-MAGE antibody preparation was tested by SDS-PAGE on a 12% polyacrylamide gel **(C)** with 2 µg of protein/well applied on gel stained with Coomassie Brilliant Blue. Murine IgE protein was confirmed by mass spectrometry in the 75 kDa band. The original gel is presented in Supplementary Figure S3. The activity of the purified anti-MAGE antibody was tested by ELISA on a plate coated with MB-mel MWG **(D)**. The mean absorbance (from 2 wells) measured at 490 nm, normalized to the absorbance of the negative sample is shown.

In addition, the epitope fit was tested by the competitive ELISA on a plate with immobilized MAGE antigen (MB-mel MWG). The dilution series of HMW-MAGE or LMW-MAGE were tested as inhibitors long with the substrates (mel, MB and NAL). It was shown that HMW-MAGE was the most potent inhibitor (Fig. S2, squares), with IC50 ~ 1.4 nmol/ml (log = 0.14). LMW-MAGE was a much weaker inhibitor (Fig. S2, triangles) with IC50 of 112.2 nmol/ml (log = 2.05). Similarly, complete inhibition of the anti-MAGE antibody reaction was achieved at a concentration of 24 nmol/ml of HMW-MAGE and at a concentration of 800 nmol/ml of LMW-MAGE. The activity of free disaccharide substrate (melibiose, Fig. 2S, circles), with IC50 of 199.5 nmol/ml (log = 2.3) was similar to LMW-MAGE and indicates epitope similarity on these two antigens. There was no anti-MAGE antibody inhibition with the free amino acid (NAL) and carrier protein (MB) (Fig. S2, asterix and rhombus, respectively) even at high concentrations, at which 100% inhibition by LMW-MAGE and HMW-MAGE was observed.

In conclusion, the obtained data suggest that the epitope structure of the anti-MAGE antibody consists a sugar and at least lysine molecule (LMW-MAGE), but the epitope is more numerous on the protein (HMW-MAGE) or may include amino acids other than lysine, which translates into a lower concentration necessary to completely block antibodies compared to the LMW-MAGE.


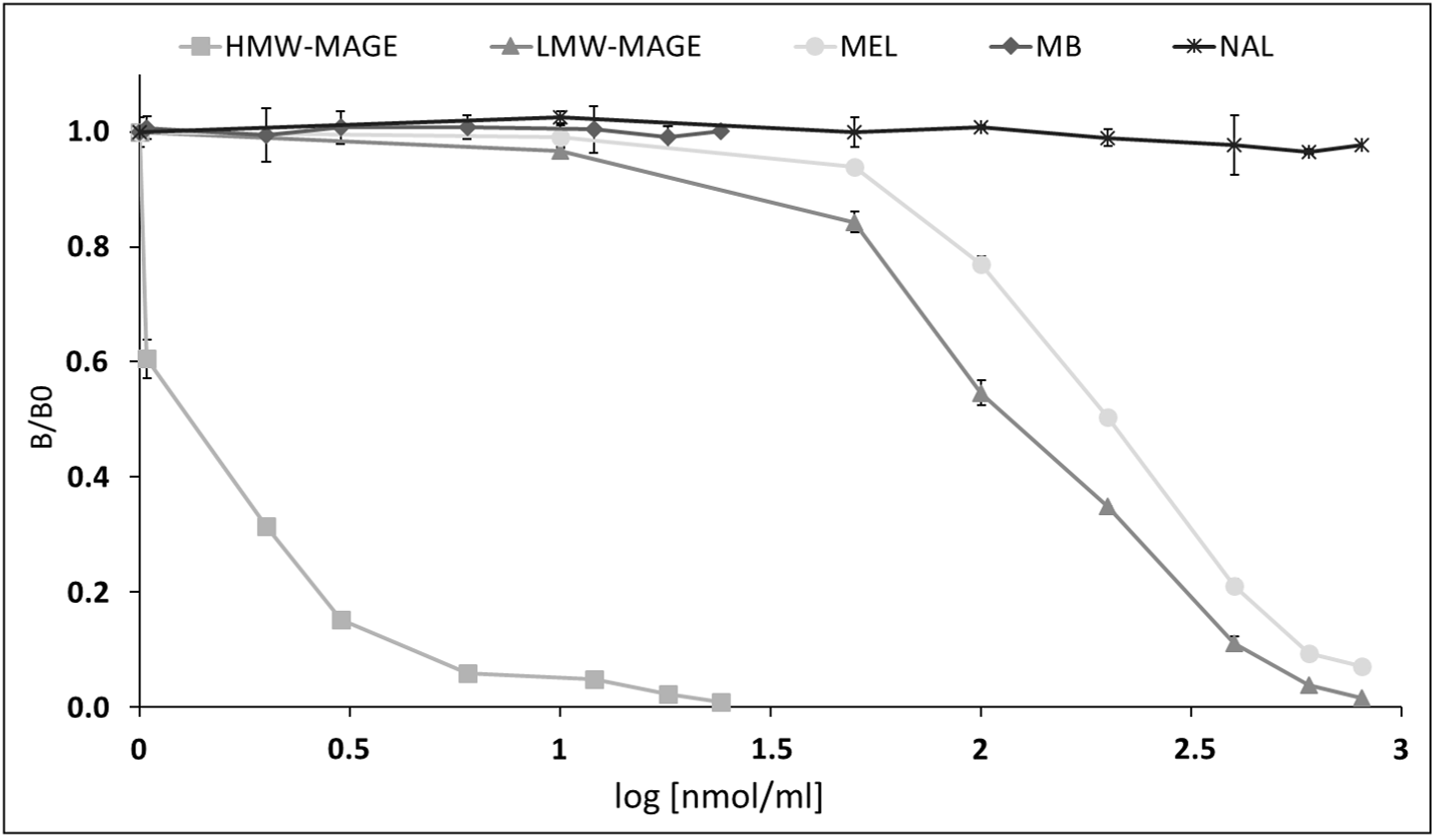


**Fig. S2.** **Testing epitope fit to the anti-MAGE monoclonal antibody.** Inhibition curves of HMW-MAGE, LMW-MAGE, MB, NAL, and mel with the purified anti-MAGE monoclonal antibody. The results after subtracting the absorbance for the negative sample (secondary antibody reaction) are presented as the ratio B/B0, where B – absorbance in the presence of inhibitor, B0 – absorbance without inhibitor (100% reaction).


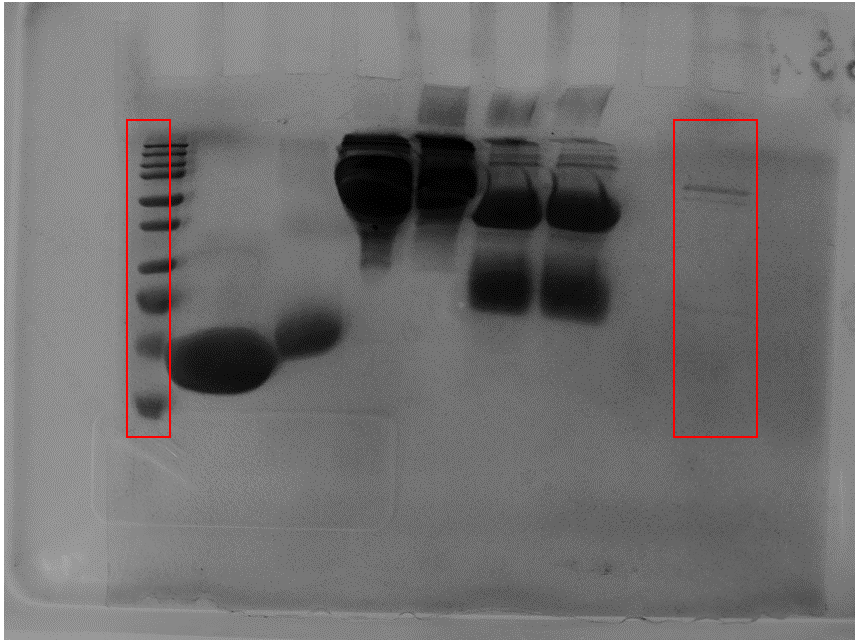


**Fig. S3. Purification and characterization of the anti-MAGE monoclonal antibody**

The original picture used in fig. S1C of the Supplementary Materials


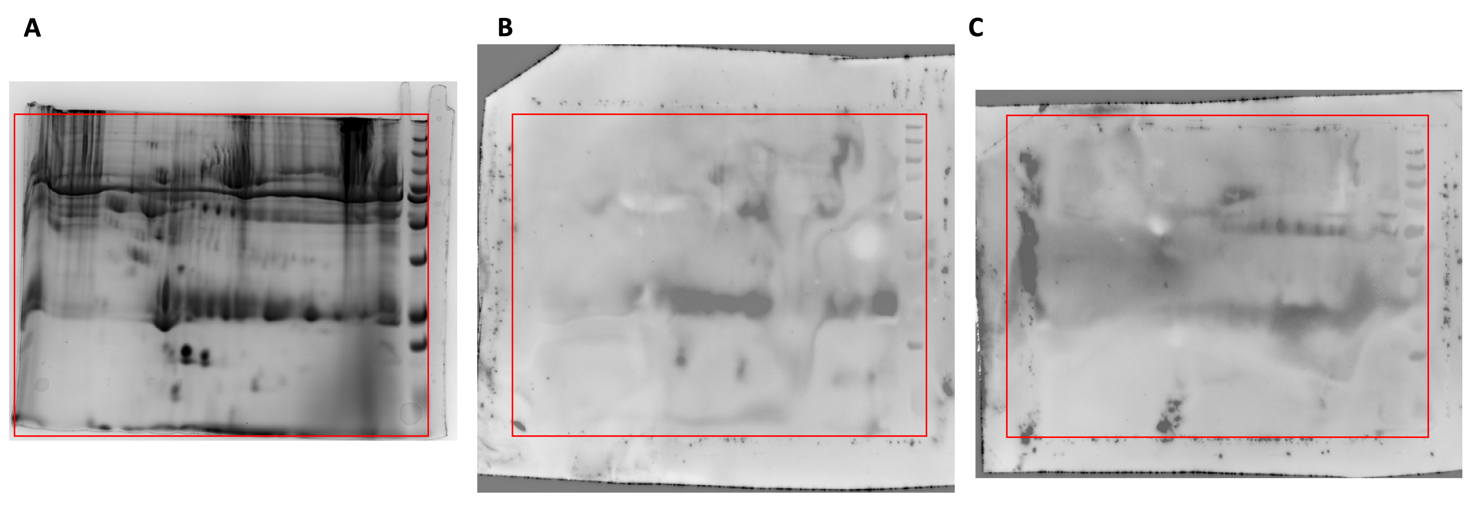


**Fig. S4. Identification of proteins glycated with MAGE**

1. The original picture used in fig. 1A, **(B)** the original picture used in fig. 1B, and **(C)** the original picture used in fig. 1C of the main manuscript


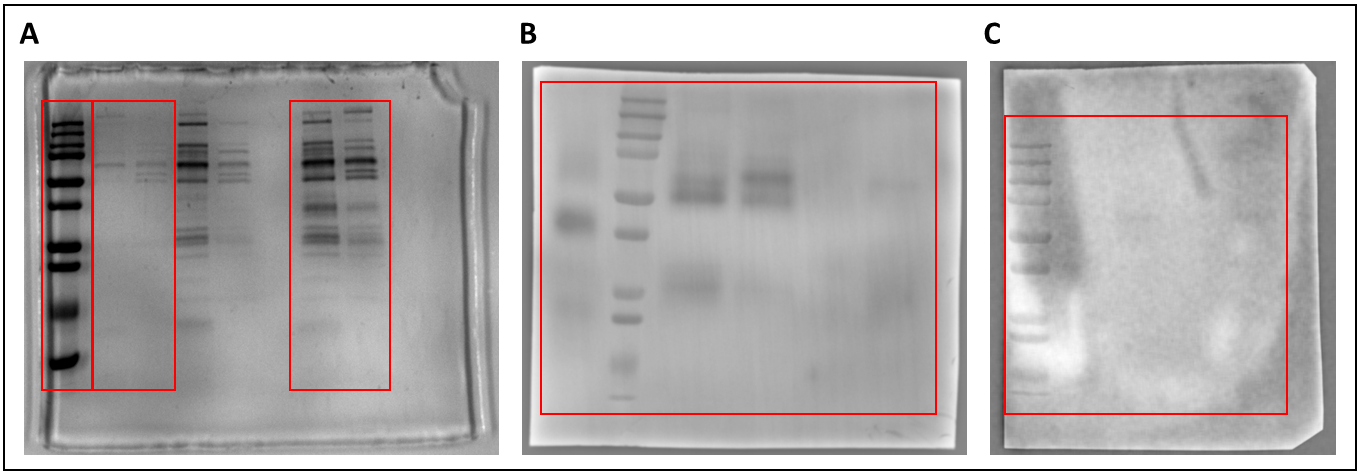


**Fig. S5. Extraction of blood proteins glycated with MAGE**

The original pictures used in fig. 2A **(A),** fig. 2B **(B)**, and fig. 2C **(C)** of the main manuscript


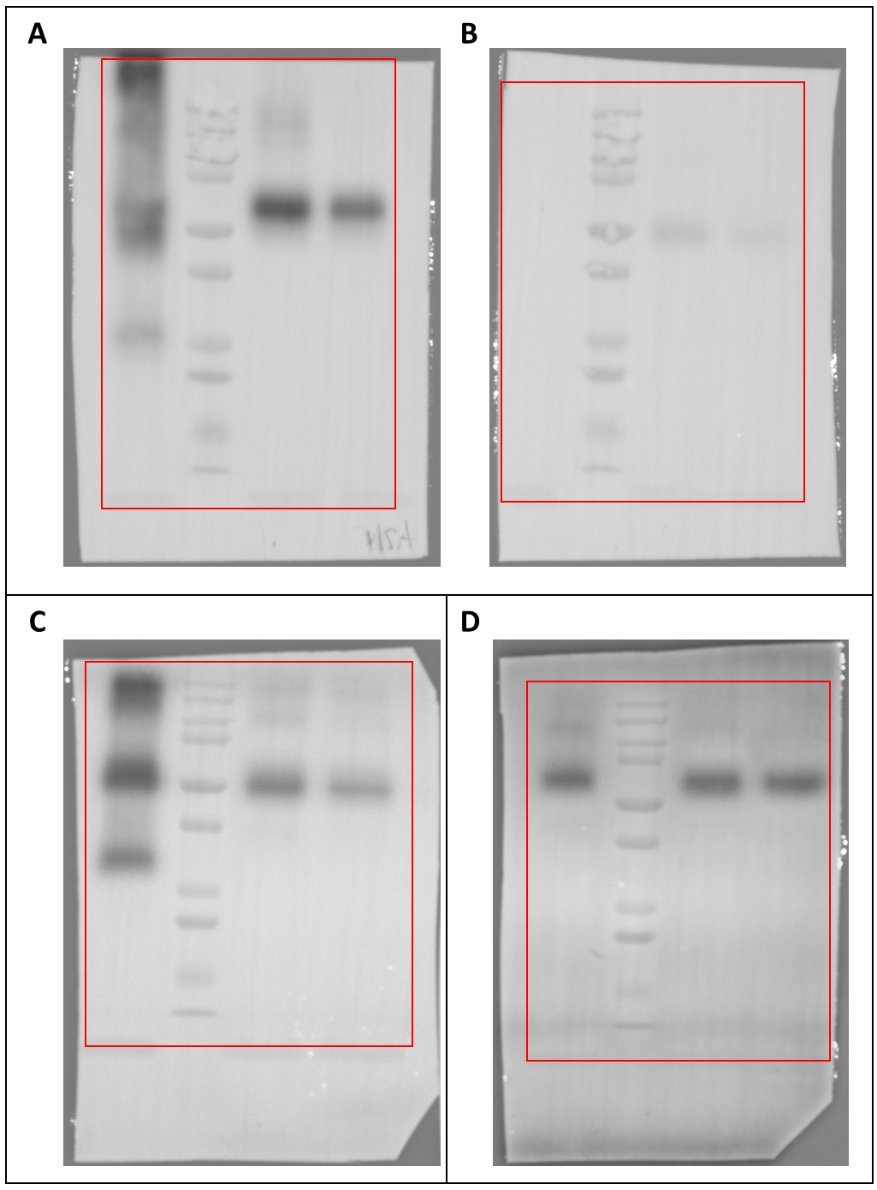


**Fig. S6. Verification of proteins extracted from human blood by WB analysis**

The original pictures used in fig. 3A **(A)**, fig. 3B **(B)**, fig. 3C **(C)**, and in fig. 3D **(D)** of the main manuscript


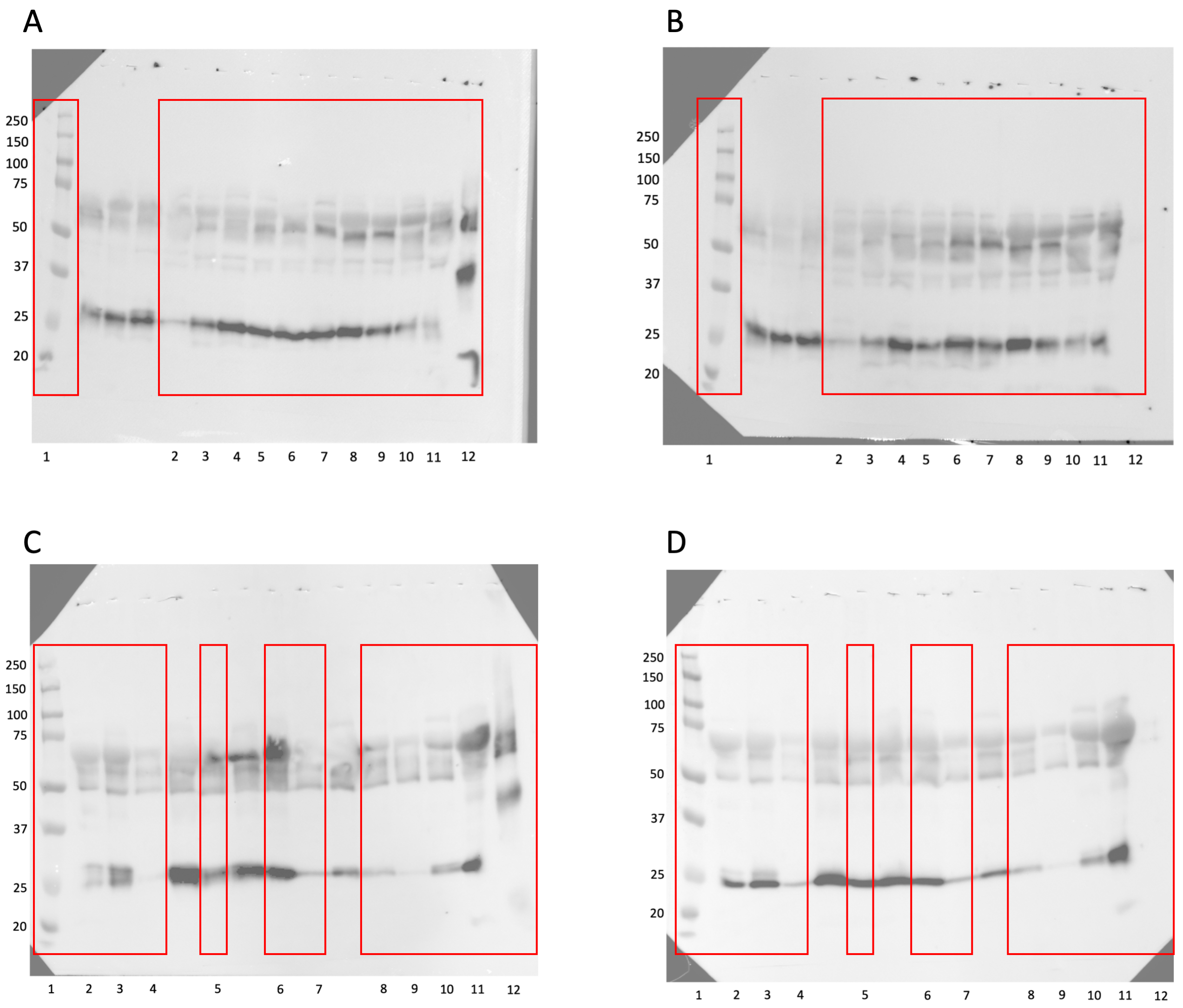


**Fig. S7. WB analysis of human body fluids with anti-MAGE monoclonal antibody**

The original pictures used in fig. 4A **(A)**, fig. 4B **(B)**, fig. 4C **(C)**, and in fig. 4D **(D)** of the main manuscript
